# Enhancement of protein translation by CRISPR/dCasRx coupled with SINEB2 repeat of noncoding RNAs

**DOI:** 10.1093/nar/gkad010

**Published:** 2023-01-30

**Authors:** Congcong Cao, Aolin Li, Chaojie Xu, Baorui Wu, Jun Liu, Yuchen Liu

**Affiliations:** Shenzhen Institute of Translational Medicine, Shenzhen Second People's Hospital, The First Affiliated Hospital of Shenzhen University, Health Science Center, Shenzhen University, Shenzhen 518035, China; Shenzhen Institute of Synthetic Biology, Shenzhen Institutes of Advanced Technology, Chinese Academy of Sciences, Shenzhen 518055, China; Department of Urology, The First Affiliated Hospital of Shenzhen University, Shenzhen Second People's Hospital, Shenzhen 518035, China; Department of Urology, The Fifth Affiliated Hospital of Zhengzhou University, Zhengzhou University, Zhengzhou 450052, Henan Province, China; Department of Urology, The First Affiliated Hospital of USTC, Division of Life Sciences and Medicine, University of Science and Technology of China, Hefei 230000, China; Urology and Lithotripsy Center, Peking University People's Hospital, Beijing 100034, China; Peking University Applied Lithotripsy Institute, Peking University, Beijing 100034, China; Shenzhen Institute of Translational Medicine, Shenzhen Second People's Hospital, The First Affiliated Hospital of Shenzhen University, Health Science Center, Shenzhen University, Shenzhen 518035, China; Shenzhen Institute of Synthetic Biology, Shenzhen Institutes of Advanced Technology, Chinese Academy of Sciences, Shenzhen 518055, China

## Abstract

The use of new long noncoding RNAs (lncRNAs) as biotechnological or therapeutic tools is still in its infancy, despite recent efforts to uncover their involvement in various biological processes including mRNA translation. An important question is whether lncRNA functional elements can be used to target translation of mRNAs of interest by incorporating the RNA-targeting CRISPR tools. The CRISPR/dCasRx-SINEB2 technology was developed in this research by coupling the sgRNA of a catalytically inactive Type VI-D Cas13 enzyme (CasRx) to an integrated SINEB2 domain of uchl1 lncRNA that promotes the translation of targeted mRNA. It has been demonstrated to be effective and adaptable in selectively increasing the expression of a variety of exogenous and endogenous proteins with a variety of functions with minimal off-target effects. dCasRx-SINEB2 is currently the sole CRISPR-related technique for translational control of gene expression, and works just as well or even better than the traditional RNAe tool under comparable conditions. Additionally, human cancer cells can be prevented from proliferating and migrating both *in vitro* and *in vivo* by dCasRx-SINEB2-targeted mRNA translation of transcripts encoding for antitumor proteins, including PTEN and P53. The present study provides an innovative protein enhancement method that will have several applications in biopharmaceuticals production and cancer research.

## INTRODUCTION

In mammals, transcription results in the production of a wide variety of transcripts, including repetitive sequences like SINEs (short interspersed nuclear elements), long noncoding RNAs (lncRNAs) and protein-coding messenger RNAs (mRNAs) (1–3). Although noncoding RNA transcripts are widely distributed in cells, little is known about their biological significance. A select handful has been identified as regulators of gene expression at the transcriptional level. Interestingly, Carrieri et al. found a new functional family of long noncoding RNAs (lncRNAs) with an overlapping antisense pattern that targets the 5′ terminal of mRNAs, which enhances the linked protein's translation (4–6). They identified anti-sense to ubiquitin carboxyterminal hydrolase L1 (AS Uchl1 or Uchl1os) as the first lncRNA that activates translation of Uchl1. Its activity is based on the combination of two domains: an embedded mouse inverted SINEB2 repeat enhancing mRNA translation (effector domain, ED) and an overlapping antisense region providing specificity for the target sense transcript (binding domain, BD). This discovery has increased our understanding of how noncoding RNAs regulate gene expression. The same team's computational research revealed that the mouse genome contains >10 000 lncRNAs with SINEB2 sequences (7). Based on a proposal, such antisense lncRNAs may be generated to improve a certain mRNA’s ability to translate. The unique antisense uchl1 lncRNA has an inverted SINEB2 motif and a 5′ pairing sequence, according to Carrieri et al. All around translation initiation codon AUG, the sense mRNA sequence and the 72 nucleotides (nt) pairing region (−42 to + 32) are fully complementary. Moreover, the SINEB2 motif improves translation by enhancing ribosome recruitment without raising mRNA levels (8). Since few technologies for activating mRNA translation have been developed, the inverted SINEB2 motif, which is critical for translation enhancement, could potentially be used to create new tools for activating translation. However, antisense RNAs’ capacity to bind target mRNAs directly may be weak, necessitating the construction of libraries to identify those antisense RNAs that have the best matching properties. Therefore, developing an easily controlled and narrowly focused mRNA translation system will significantly influence the development of biopharmaceuticals and molecular studies.

One of the most adaptable techniques for controlling genomic DNA is the CRISPR-Cas9 system, which can not only edit gene sequences, but also concurrently activate or suppress the transcription of endogenous genes. In addition to regulating DNA, CRISPR technology can also be used to regulate RNAs (9–13). CRISPR-associated nuclease Cas13 technology advancements have made it possible to edit mRNA precisely and examine RNA alterations during biological processes (14). The Cas13 enzymes bind and cleave single-stranded RNA molecules complementary to their guide RNAs (15). Cas13 family proteins include Cas13a (1250 aa), Cas13b (1150 aa), Cas13c (1120 aa) and Cas13d (930 aa), which have been reported to have considerable RNA knockdown and minimal off-target effects (16). In combination with a nuclear localization sequence (NLS), the RfxCas13d (CasRx), the smallest known enzyme in the Cas13 family, exhibits the best-targeted knockdown performance (16–18). Previous strategies that tethering catalytically inactive CasRx (dCasRx) to m^6^A writers or erasers could manipulate specific m^6^A sites targeted by relevant Cas13 guide RNAs (19). However, the CRISPR-dCasRx-based device can still not regulate the translation of mRNA, which is the final step of gene expression and directly determines the intensity of gene expression. Even though CRISPR-based trancriptional factors can control gene transcription directly, many genes’ levels of mRNA and protein expression have been shown to not always be proportionate (20,21). Although gene overexpression vector or CRISPR-mediated transcription activation can significantly increase the mRNA expression level of the gene, its protein level often only increases by 2–3 times (22).

In order to solve the above mentioned problems, we designed and developed a CRISPR-dCasRx-based method to target protein translation of specific mRNA in the current study. A synthetic RNA contruct was produced by linking the catalytically inactive Cas13Rx-sgRNA with the SINEB2 element for translational stimulation of mammalian protein-coding genes. CRISPR-dCasRx-SINEB2 effectively manipulated protein activation within endo-transcripts and resulted in negligible off-target changes in both cancerous and healthy mammalian cells. The small size of dCasRx-based translational regulators allow both dCasRx and crRNA-SINEB2 constructs to be packaged into one single adeno-associated virus (all-in-one AAV) vector to treat cells that are difficult to transfect using other strategies. In addition, much efforts have been made to improve the efficiency of CRISPR-dCasRx-SINEB2 in different situations and to expand its scope for broad applicability. We used the CRISPR-dCasRx-SINEB2 system on anti-oncogenic targets and effectively inhibited cell migration and proliferation, indicating that this designed tool has biotechnological potential. These studies offer a powerful tool for *in vivo* gene-specific mRNA translation enhancement and a novel class of RNA-based medication for molecular treatment. In addition, this is the first CRISPR-based technique to initiate gene translation to the best of our knowledge.

## MATERIALS AND METHODS

### Plasmids construction and AAV production

The plasmid dCasRx-sgRNA-SINEB2 was created by inserting the dCasRx-crRNA sequence fused with the SINEB2 domain sequence into the same backbone. Using NCBI BLAST (https://blast.ncbi.nlm.nih.gov/Blast.cgi), the original sgRNAs were examined to prevent undesired mRNA off-target binds in the human genome. The gRNA sequence is ∼20 nt in length and its binding site is located around the translation initiation codon AUG. Also, the targeted sequences were chosen at GC% = 40–60% and filtered for off targets. The uniquely aligned sequences were further aligned to the transcriptome. Each sgRNA intended complementary DNA (cDNA) sequence was created, and it was then introduced into the appropriate plasmid (Syngentech Co., Ltd, Beijing, China) that expressed both dCasRx and crRNA. Plasmid pcDNA 3.1 was used as the negative control DNA. The plasmids mentioned above and their related sequences are listed in the Supplementary material (Tables S1–S3). AAV-dCasRx-SINEB2 were packaged by transfection into HEK293T cells using polyethylenimine (PEI) (50 μg/mg). Viruses were harvested, purified, and concentrated 3–7 days after transfection.

### Cell culture

In Dulbecco's modified Eagle's medium (DMEM, Gibco, USA) with 0.1 mg/ml streptomycin (Biodee, China), 100 units/ml penicillin (Biodee, China) and 10% FBS (foetal bovine serum), HEK293T, T24 and HeLa cells were cultivated. In RPMI 1640 medium (DMEM) (Gibco, USA) with 0.1 mg/ml streptomycin (Biodee, China), 100 units/ml penicillin (Biodee, China) and 10% foetal bovine serum (FBS), HepG2 and 5637 cells were cultured. All cultures were incubated at 37°C in 5% CO_2_ and 95% humidified air. Cells were divided using TrypLE Express (Life Technologies) at 80–90% confluency and passaged at a 1:2 ratio. These cell lines were not otherwise validated; they were obtained from the American Type Culture Collection (Manassas, VA, USA) directly.

### Cell transfection

The plasmids were extracted from *Escherichia coli* using the EZN-A Fastfiler Endo-free Plasmid Maxiprep kits from Omega in Norcross, Georgia, USA. According to the manufacturer's instructions, cells were transfected with various plasmids using lipofectamine-3000 (Invitrogen, USA). HEK293T cells were transfected with pEGFP vector and Negative Control, RNAe-EGFP or dCasRx-SINEB2-EGFP constructs. T24, HepG2 and HeLa cells were transfected with pEGFP vector and dCasRx-SINEB2-NT or dCasRx-SINEB2-EGFP. T24 and 5637 cells were transfected with dCasRx-SINEB2-NT, dCasRx-SINEB2-PTEN or dCasRx-SINEB2-P53 constructs. Before being tested in 6-well assays, cells were transfected for 48 h with dCasRx plasmids (1 μg) and sgRNA (1 μg).

### Quantitative real-time PCR

The RNAeasy™ RNA Isolation Kit (Beyotime Biotechnology, China) was utilized to extract total RNA from cells transfected with various vectors described as before. Utilizing the BeyoRTTM II cDNA Synthesis Kit (Beyotime Biotechnology, China), cDNA was created. GAPDH was used as the endogenous control, and SYBR Green qPCR MasterMix (Takara, Dalian, China) was utilized to examine the mRNA expression. The primer sequences for GAPDH, GFP, SINEB2, PTEN, P53, PTEN and TIMP-1 are presented below, with 5′ to 3′ lengths;

GAPDH-F: TCCCATCACCATCTTCCA

GAPDH-R: CATCACGCCACAGTTTCC

GFP-F: ACGACGGCAACTACAAGACC

GFP-R: TTGTACTCCAGCTTGTGCCC

SINEB2-F: CAGTGCTAGAGGAGGTCAGAAGA

SINEB2-R: GGAGCTAAAGAGATGGCTCAGCACTT

P53-F: CCTCAGCATCTTATCCGAGTGG

P53-R: TGGATGGTGGTACAGTCAGAGC

PTEN-F: TCCCAGACATGACAGCCATC

PTEN-R: TGCTTTGAATCCAAAAACCTTACT

TIMP-1-F: AGTCATCAGGGCCAAGTTCG

TIMP-1-R: CACAGCCAACTTTCTCGCAC

### Western blot

For western blot analysis, cell pellets were resuspended in sample buffer, quickly sonicated, boiled and loaded onto polyacrylamide gels. Anti-GFP rabbit polyclonal antibodies (Life Technologies) and anti-P53 (Abcam) were both used at a dilution of 1:1000. Anti-GAPDH (Invitrogen) was diluted at a ratio of 1:5000, and anti-PTEN (Invitrogen) and anti-TIMP1 (Abcam) were diluted at a ratio of 1:500. ECL was used in conjunction with anti-mouse-HRP or anti-rabbit-HRP (Dako) for detection (GE Healthcare). The Alliance LD2-77WL system (Uvitec, Cambridge) was employed for image detection.

### RNA-immunoprecipitation

A modified version of the Abcam protocol (https://www.abcam.com/epigenetics/rna-immunoprecipitation-rip-protocol) was used for the RIP assay. HEK293T cells were seeded in 10-cm plates and then transfected with plasmid as indicated above. The cells were subjected at 254 nm UV light with 300 mJ/cm^2^ and the nucleus and cytoplasmic fractions of cells were isolated the next day. Nuclear pellets were broken up by sonication 5 cycles (ON for 30 s, OFF for 30 s) using a Bioruptor Pico device. The target protein antibodies were used to immunoprecipitate each lysate for an entire overnight period at 4°C. After adding Protein A/G magnetic beads from Invitrogen to bind the target antibodies, unbound antibodies were removed using a series of washing steps. PTBP1 and HNRNPK were immunoprecipitated in each nuclear or cytoplasmic fraction using the anti-hnRNPK mouse monoclonal antibody (3C2)-ChIP Grade (ab39975, Abcam) and the anti-PTBP1 mouse monoclonal antibody (32-4800, Thermo Fisher Scientific). The lysates were treated with protease K overnight at 55°C to filter the RNA, which was then extracted using chloroform (WAKO) and Trizol (Thermo Fisher Scientific, Germany) extraction method. The amount of RNA was measured using qRT-PCR.

### Cell proliferation assay

Cell proliferation was determined using the CCK 8 assay kit (Transgen, China). 96-well plates were seeded with 3 × 10^3^ cells per well. The cells were cultured for 24, 48, 72 and 96 h before being treated with CCK8 at 37 °C for 3 h. The absorbance at 450 nm was measured using a microplate reader.

### Wound healing migration assays

The wound healing migration experiments were carried out as described. Cells were planted at a density of 5 × 10^5^ cells/well in a six-well chamber slide. A sterile 10 μl pipette tip was used to scratch the center of the slides. After 48 h of incubation, photos were taken to assess the gap's closure. The wound closure was measured using the following formulae:


$$\begin{equation*}{\rm{Wound\;closure}} = \frac{{\left( {{\rm{area\;of\;the\;gap\;}}\left[ {0{\rm{h}}} \right] - {\rm{the\;area\;of\;the\;gap\;}}\left[ {48{\rm{h}}} \right]} \right)}}{{{\rm{area\;of\;the\;gap\;}}\left[ {0{\rm{h}}} \right]}}\;\end{equation*}$$

### Cell apoptosis assay

On a 12-well plate, 2 × 10^5^ vector-transfected cells per well were inoculated when they were 70–80% confluent. The caspase-3 ELISA assay kit (Hcusabio, China) was used to identify cell apoptosis after 48 h. Because it can control the degradation of DNA or cytoskeletal proteins, caspase-3 is a marker for inflammation and apoptotic signaling. Each experiment was performed in triplicate,

### Xenograft model

Female BALB/c nude mice (aged 4–5 weeks) were randomly separated into four groups of six (standard housing settings) and maintained under specific-pathogen-free conditions. The right flanks of the mice were injected subcutaneously with 5 × 10^6^ transfected bladder cancer cells. Volumes of tumors were measured weekly. Four weeks after receiving an injection, the mice were sacrificed humanely under anesthesia. Isolation of subcutaneous tissue and measurement of the volume and mass of the dissected tumors were then performed. All experimentations were authorized by the Shenzhen Second People's Hospital's animal management committee, and all experimental techniques and animal care complied with the institution's ethical norms for animal experimentation.

### Experimental metastatic mouse model

Male B-NDG mice (5 weeks old) were treated with 1 × 10^5^ T24 bladder cancer cells expressing luciferase suspended in 200 μl of PBS (phosphate-buffered saline). The mice were administered isoflurane anesthesia and intraperitoneal injections of d-luciferin sodium salt (150 mg/kg) were performed four weeks later. The *in vivo* Xenogen IVIS system (PerkinElmer, Boston, MA, USA) was used to count the bladder cancer cells. Using the Living Image 4.3.1 program (PerkinElmer/Caliper), the total flux of photons per second for the lung region was determined.

### Statistical analysis

The data were shown as the mean ± standard deviation (SD). GraphPad Prism 8.0 software (GraphPad, San Diego, CA, USA) and SPSS 20.0 (IBM, SPSS, Chicago, IL, USA) were used for the statistical analyses. Significant differences between the two groups were analyzed using the two-sided Student's *t*-test and two-tailed Mann–Whitney *U*-test, and that between multiple groups was measured using one-way analysis of variance followed by Bonferroni post hoc tests. A value of *P* < 0.05 was considered statistically significant for any differences. *P* values are denoted in figures or figure legends by *P < 0.05; **P < 0.01 and ***P < 0.001.

## RESULTS

### Design of a targeted mRNA translation system named CRISPR/dCasRx-SINEB2

**Figure 1.**
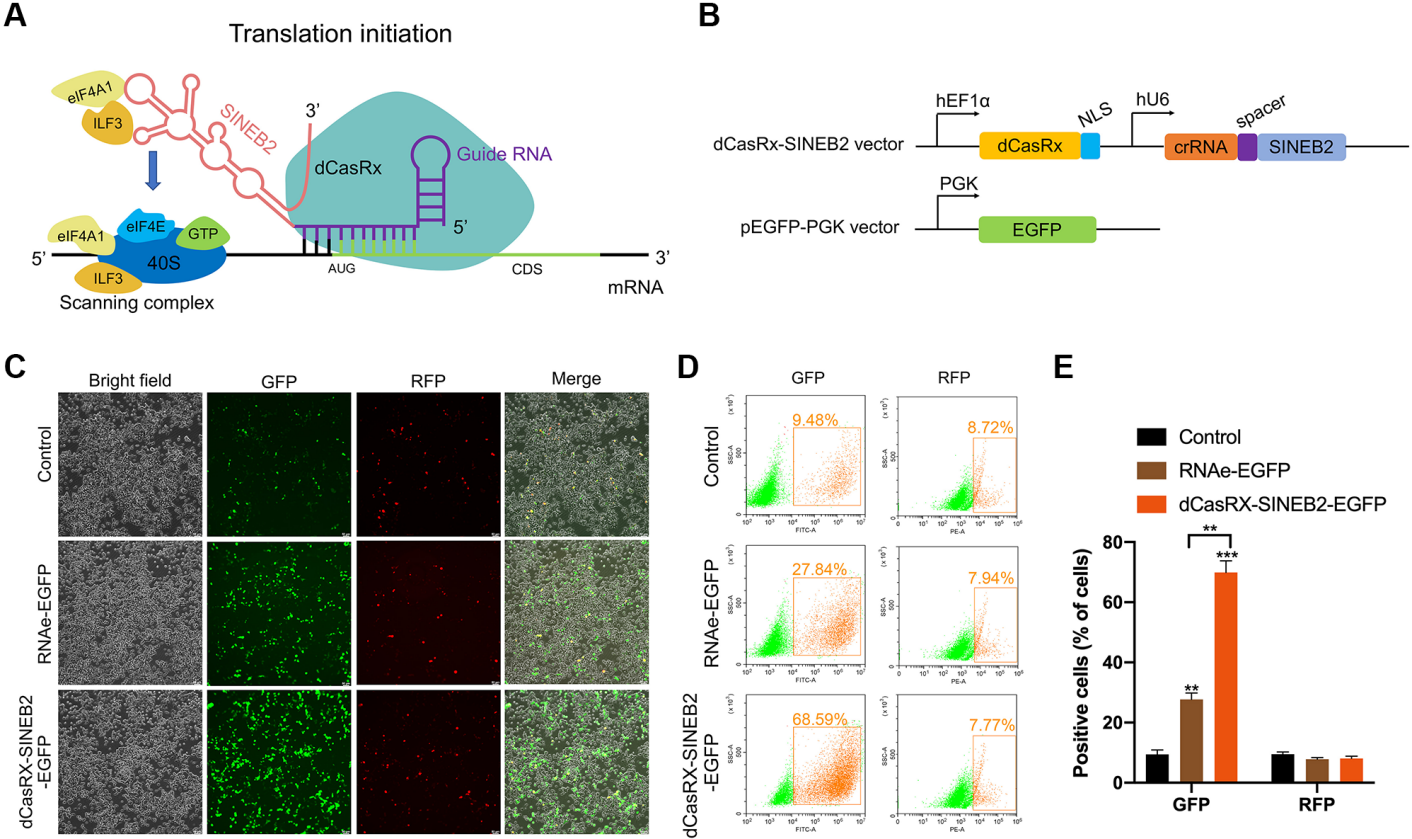
A schematic illustration of CRISPR/dCasRx-SINEB2 development. (**A**) Schematic diagram of the sgRNA-guided dCasRx-SINEB2 mRNA translation activation system that consists of two parts: one part contains sgRNA targeting the translation initiation region around the AUG start codon of the gene of interest; another part contains inverted SINEB2 element that confers protein synthesis activation via ribosome recruitment. (**B**) Graphical view of the CRISPR/dCasRx-SINEB2 construct and its target EGFP. (**C**) The impact of dCasRx-SINEB2 was evaluated by fluorescence spectroscopy in HEK293T cells co-transfected with pEGFP-C1,pRFP and control plasmid, pRNAe-EGFP plasmid, or dCasRx-SINEB2-EGFP plasmid. (**D**, **E**) The results depicted a flow cytometry analysis of plasmid-transfected EGFP-positive cells. Statistical data from independent triplicate experiments are shown as mean ± SD. The statistical significance was defined as **P < 0.01, ***P < 0.001.

An optimized gene-specific translational regulatory platform should show specificity and efficiency in binding to target sites. In order to accomplish this, we combined the dCasRx-gRNA with the SINEB2 element of the long noncoding RNA uchl1, which can improve the target mRNA’s translation (Figure 1A) (4). We established a recombinant dCasRx-SINEB2-EGFP construct targeting EGFP mRNA to verify the effectiveness of translation enhancement (Figure 1B and Supplementary Figure S1). HEK293T cells were co-transfected with dCasRx-SINEB2-EGFP, pEGFP-C1 and pRFP, and then living cells were studied using a fluorescent microscope and fluorescence flow cytometry (Figures 1C and D). The results showed that dCasRx-SINEB2-EGFP was sufficient to enhance GFP expression without affecting RFP. Additionally, RNAe is a traditional tool for universal protein expression enhancement that was created by adding the SINEB2 repeat to an antisense noncoding RNA (23). Our results revealed that the dCasRx-SINEB2-EGFP group had a substantially larger percentage of EGFP-positive cells than the RNAe-EGFP group (Figure 1E). Then, we designed three control groups to compare their activation efficiency with that of dCasRx-gRNA-SINEB2. The results showed that the percentage of EGFP-positive cells was significantly higher in the dCasRx-gRNA-SINEB2 group compared with the gRNA-SINEB2 group without dCasRx, the dCasRx group without gRNA-SINEB2, and the dCasRx-gRNA group without SINEB2, respectively (Supplementary Figure S2). Finally, we wanted to systematically explore a library of different target locations to determine good design principles for constructing CRISPR/dCasRx-SINEB2. For this, we compared the fluorescence of cells containing crRNAs at different targeting locations. The results demonstrated that guide crRNAs targeting GFP-4 and GFP-5, which located around the AUG start codon significantly enhanced the activation effect of GFP coupling with dCasRx-SINEB2 (Supplementary Figure S3).

### CRISPR/dCasRx-SINEB2-EGFP enhances EGFP mRNA translation through ribosome recruitment

**Figure 2.**
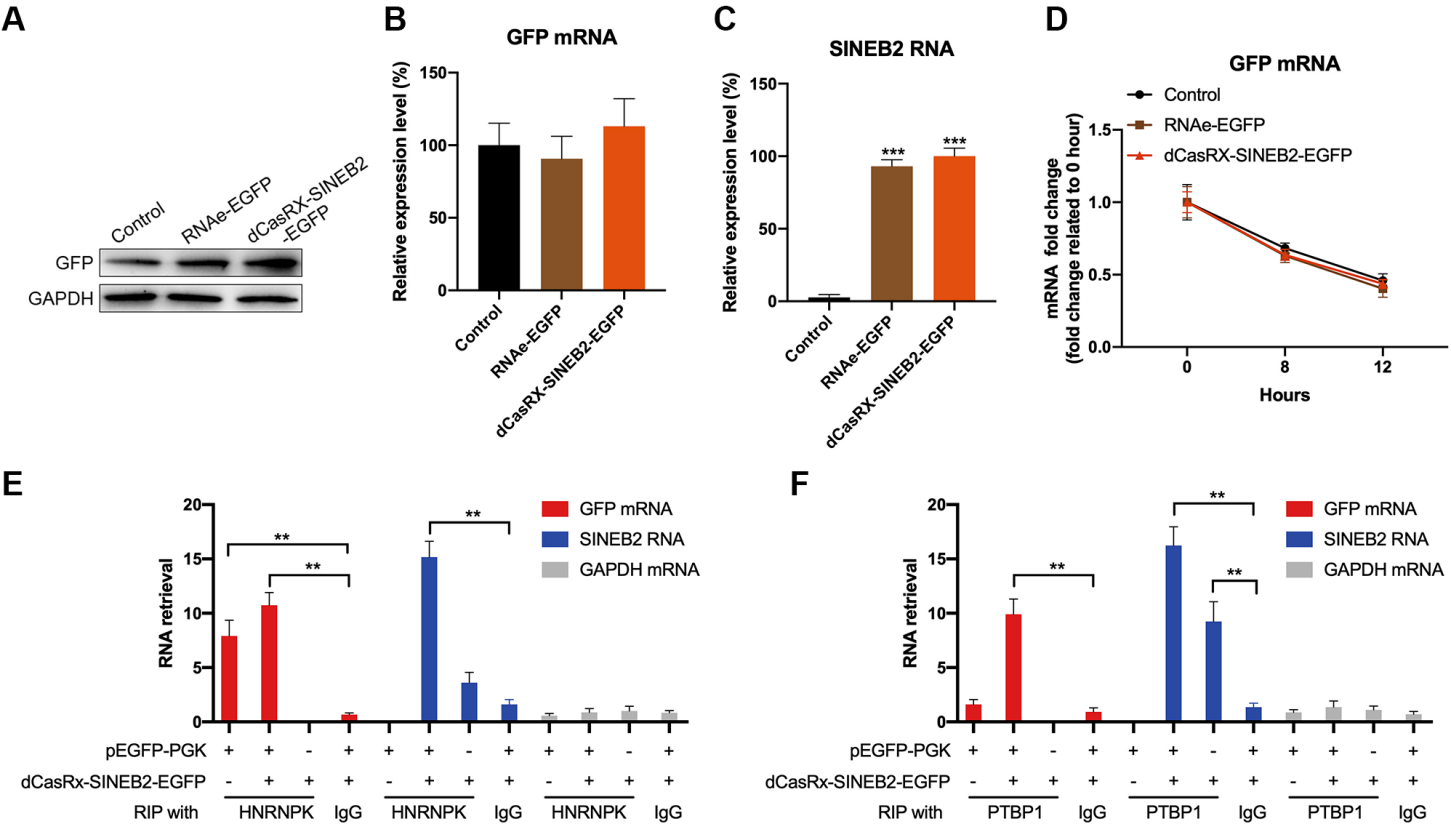
In HEK293T cells, CRISPR/dCasRx-SINEB2-EGFP increases EGFP mRNA translation. (**A**) HEK293T cells co-transfected with the pEGFP-C1 plasmid, the control plasmid, the pRNAe-EGFP plasmid, or the dCasRx-SINEB2-EGFP plasmid underwent a Western blot analysis to determine the degree to which EGFP expression was increased. (**B**, **C**) qRT-PCR was utilized to determine the relative mRNA levels of GFP and SINEB2 using the identical constructs described above. (**D**) Actinomycin D (ActD) was added to the HEK293T cell culture media for 0, 8 or 12 h to suppress mRNA synthesis after transfection with pEGFP, pRNAe-EGFP, or dCasRx-SINEB2-EGFP. According to the results of qRT-PCR, dCasRx-SINEB2 did not affect the stability of mRNA. (**E**, **F**) RNA immunoprecipitation (RIP) in HEK293T cells with antibodies against HNRNPK (**E**), PTBP1 (**F**) or Isotype IgG (isotype immunoglobulin G) served as the negative control. After co-transfection with the pEGFP and dCasRx-SINEB2-EGFP vectors or after transfection with either vector alone, cell lysates were examined. *n* = 3; the statistical data of three independent experiments are expressed as mean ± SD. The **P < 0.01 and ***P < 0.001 denotes the statistically significant values.

To confirm the translational up-regulation activity of dCasRx-SINEB2 constructs, we prepared cell lysates to accurately measure the increase of target protein levels with western blot. According to the findings, after HEK293T cells were transfected with dCasRx-SINEB2-EGFP, there was a significant induction. In addition, we also obtained an induction in the cells treated with RNAe-EGFP, which was less efficient than dCasRx-SINEB2-EGFP (Figure 2A, Supplementary Figure S4A). The number of GFP-positive and total number of cells were normalized to determine the transfection efficiency of this study (Figure 1C-E), and the expression of GFP mRNA and SINEB2 RNA was assessed (Figures 2B and C). Results from qRT-PCR on HEK293T cells transfected with dCasRx-SINEB2-EGFP revealed that this construct had no impact on the target EGFP’s mRNA level (Figures 2B). Actinomycin D (ActD) was employed to inhibit transcription in a different experiment, and the results of qRT-PCR revealed that dCasRx-SINEB2-EGFP likewise did not affect the rate at which EGFP mRNA degraded (Figure 2D). These findings proved that the effect of dCasRx-SINEB2 occurs post-transcriptionally.

HNRNPK and PTBP1 have been recognized as important RNA binding proteins by previous research (8). These proteins aid in creating translational initiation complexes and distribution of SINEUP RNA inside the cell, thus enhancing the translation of the target mRNA. We performed an RNA RIP (immunoprecipitation) experiment of RNA-protein interactions with HNRNPK and PTBP1 proteins in HEK293T cells to confirm the function of HNRNPK and PTBP1 interactions in dCasRx-SINEB2-EGFP. The results showed that EGFP mRNAs were pulled down with HNRNPK (Figure 2E) and dCasRx-SINEB2-EGFP RNAs were pulled down with PTBP1 (Figure 2F).

In order to validate that, as suggested by Carrieri et al., dCasRx-SINEB2 operates by attracting polysomes to the target mRNA, qRT-PCR and polysome profiling were performed with earlier mentioned co-transfected HEK293T cells (Supplementary Figure S5). When cells were transfected with dCasRx-SINEB2-EGFP, the results indicate that EGFP mRNAs preferred to bind to heavier polysomes (*n* = 7 or higher) (Supplementary Figure S5B). At the same time, the pattern of binding for GAPDH mRNAs (an internal control) did not change (Supplementary Figure S5A). HEK293T cells with stable EGFP expression were transfected with dCasRx-SINEB2-EGFP and negative control plasmid DNA, respectively. Following an earlier study, the proteomics analysis was carried out using quantitative mass spectrometry (24). Except for the increased EGFP expression in the experimental condition, there was no significant difference between these two groups could be observed (Supplementary Figure S5C), which means that dCasRx-SINEB2 does not affect non-targeted cellular protein expressions in general. The information about the constructed plasmids were listed in Supplementary Figures S6–S8. When all of these results are put together, they show that dCasRx-SINEB2-EGFP RNAs work with HNRNPK and PTBP1 to recruit ribosome subunits to help form translational initiation complexes, which are needed for the first step of translation.

### *In vitro* assessment of different cell line's responses to CRISPR/dCasRx-SINEB2

**Figure 3.**
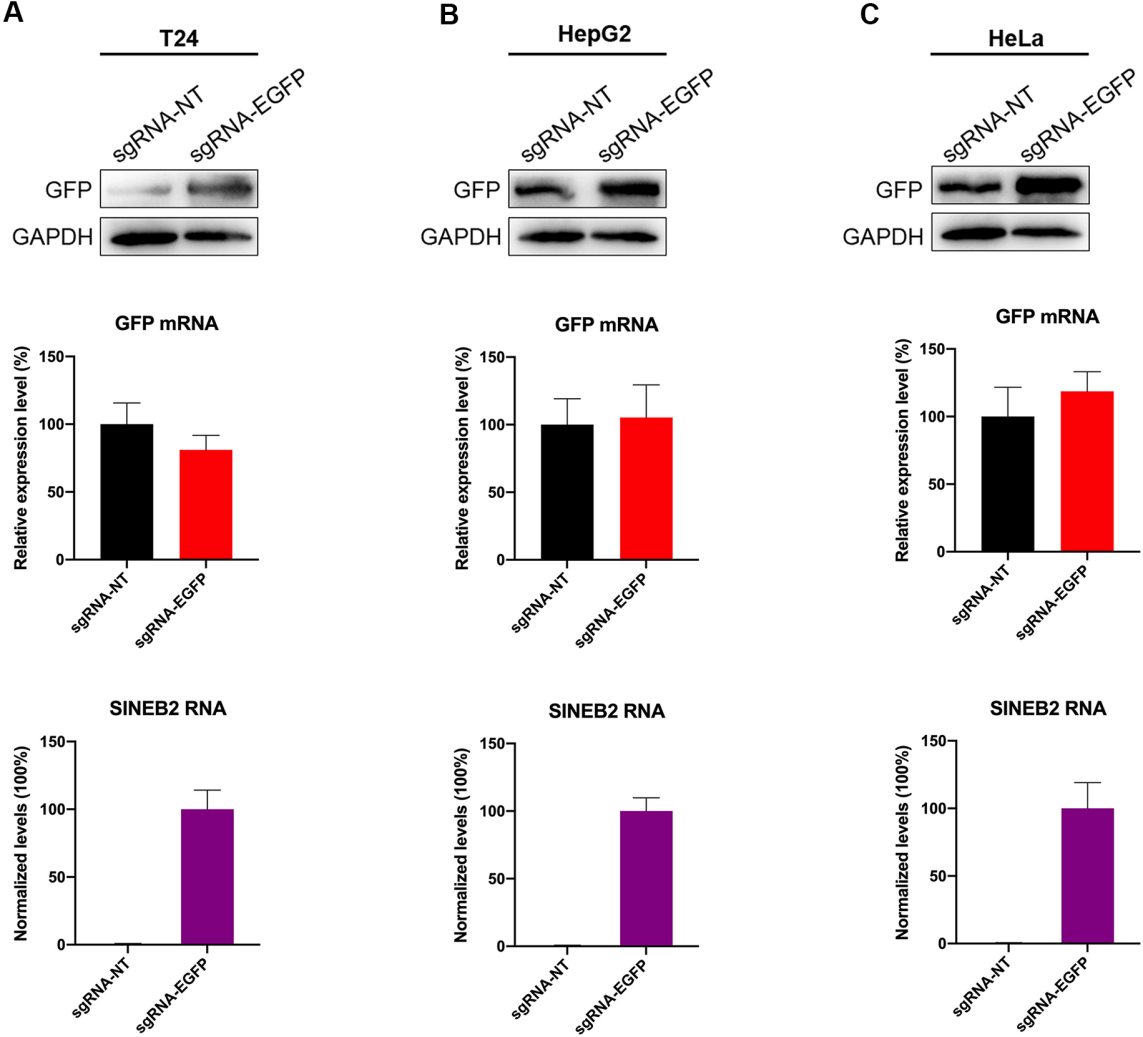
The action of CRISPR/dCasRx-SINEB2 in several cell lines. (A–C) T24 (**A**), HepG2 (**B**) and HeLa (**C**) cells were transfected using dCasRx-SINEB2-NT and dCasRx-SINEB2-EGFP. Following transfection, cells were lysed and processed to measure the amounts of GFP protein (top) and mRNA (middle) 24 or 48 h later in HeLa or T24 and HepG2 cells. An anti-GFP antibody was used in the western blot analysis. As a loading control, GAPDH was used. Western blot images adjusted to GAPDH and relative to empty control samples were employed to calculate fold-induction. Using the relevant primers, qRT-PCR was used to evaluate the expression of SINEB2 and the quantity of GFP mRNA. The statistical data of three independent experiments are expressed as mean ± SD.

Mammalian cell cultures are frequently used as a common model to study the cell factories and the molecular mechanisms of gene functions that produce therapeutic proteins *in vitro*. We therefore studied the efficacy and repeatability of dCasRx-SINEB2 in different cell lines *in vitro*, taking into account the versatility of dCasRx-SINEB2 technology and its prospective uses in molecular biology investigations, protein synthesis, and therapies. Due to their extensive use in therapeutic protein production and cell biology, we chose bladder cancer T24 cells, hepatocellular carcinoma HepG2 cells, and epithelial carcinoma HeLa cells for this purpose. We utilized dCasRx-SINEB2-EGFP to boost GFP protein levels in temporary overexpression assays. We calculated the fold changes in protein levels encoded by the targeted mRNAs in the presence or absence of dCasRx-SINEB2 with mRNA amounts kept constant. T24, HepG2 and HeLa cells supported dCasRx-SINEB2 activity, with an significant induction, according to Western blot (Figure 3A–C, Supplementary Figure S4B–D) and flow cytometry (Supplementary Figure S9) analysis. These findings collectively show that dCasRx-SINEB2 can be employed to up-regulate desired proteins *in vitro* in various cell systems.

Previous studies has found that there were no highly significant correlations between many genes’ mRNA and protein expression levels (20,21). For example, matrix metalloproteinases (MMPs) and their inhibitors (TIMPs) play important roles in dentine formation, caries progression and hybrid layer degradation. Another limited analysis, of the three genes MMP-2, MMP-9 and TIMP-1 in human prostate cancers, showed no significant relationship between mRNA and protein expression levels (25).Therefore, we compared the protein activation effect of our CRISPR/dCasRx-SINEB2 with that of CRISPR activation (CRISPRa), which boosts transcription of a target gene and the overexpression vector. The results showed that the protein level of TIMP-1 was significantly increased after transfected with CRISPR/dCasRx-SINEB2-TIMP-1, while this effect was attenuated in CRISPRa and pcDNA-TIMP-1 groups (Supplementary Figure S10A, B). Although the TIMP-1 mRNA expression was highly activated after transfected with CRISPRa and pcDNA-TIMP-1 (Supplementary Figure S10C), the protein level of TIMP-1were not elevated accordingly.

### CRISPR/dCasRx-SINEB2 increases endogenous tumor suppressor genes protein expression in bladder cancer cells

**Figure 4.**
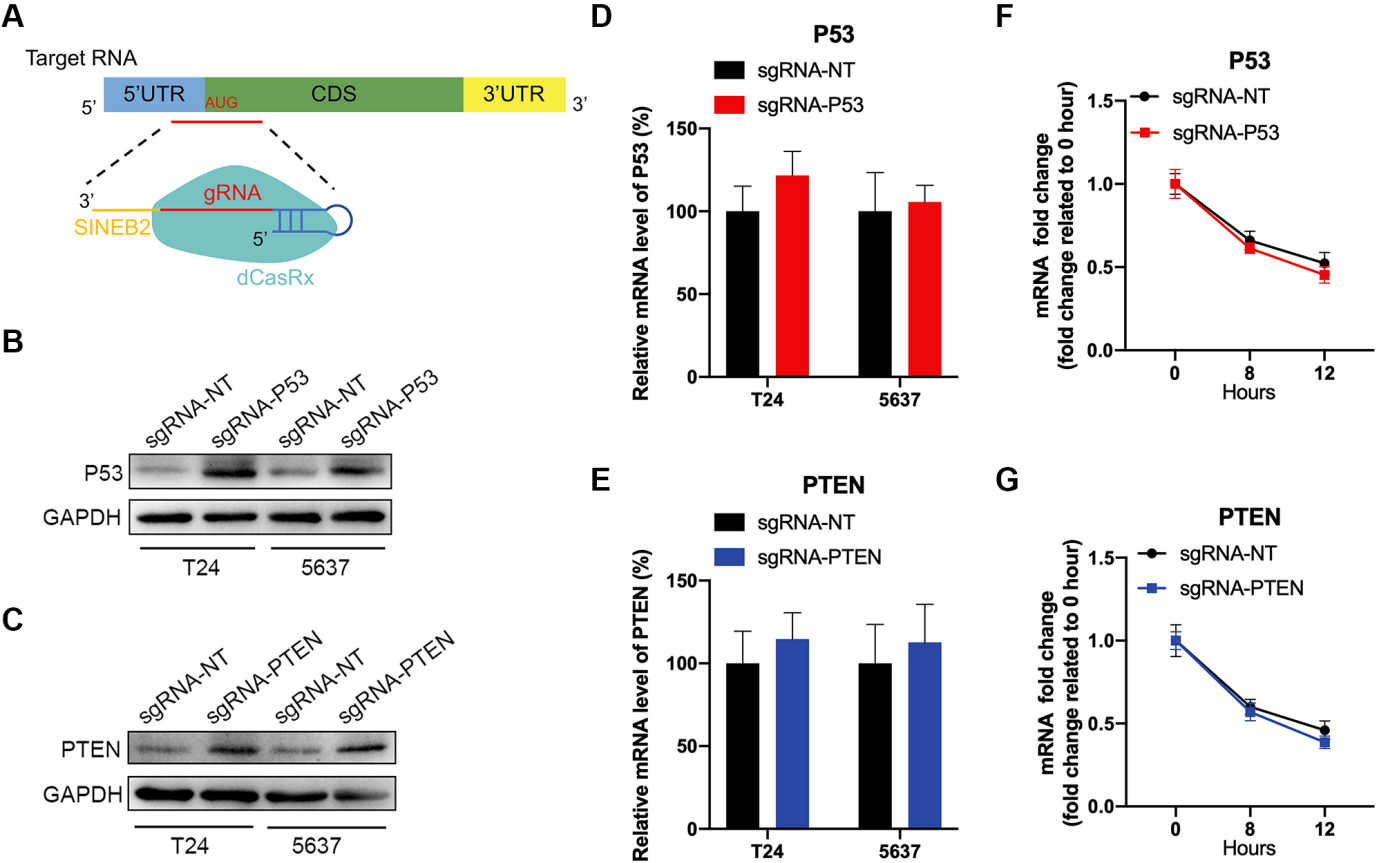
Wide applications of CRISPR/dCasRx-SINEB2 in bladder cancer cells. (**A**) Schematic representation of dCasRx-SINEB2 constructs targeting endogenous tumor suppressor genes. (**B**, **C**) P53 (B) and PTEN (C) protein expression levels were detected after being transfected with dCasRx-SINEB2-NT, dCasRx-SINEB2-P53 or dCasRx-SINEB2-PTEN in T24 and 5637 bladder cancer cells by western blot. (**D**, **E**) P53 (D) and PTEN (E) mRNA expression levels were detected after being transfected with dCasRx-SINEB2-NT, dCasRx-SINEB2-P53 or dCasRx-SINEB2-PTEN in T24 and 5637 bladder cancer cells by qRT-PCR. (F, G) Actinomycin D (ActD) was applied to the bladder cell culture medium for 0, 8 or 12 h after transfection with dCasRx-SINEB2-NT, dCasRx-SINEB2-P53 or dCasRx-SINEB2-PTEN to inhibit mRNA synthesis. The qRT-PCR assay revealed that dCasRx-SINEB2 did not affect P53 (**F**) or PTEN (**G**) mRNA stability. Data are representative of >3 independent replicates.

We then investigated whether dCasRx-SINEB2 might be used consistently to improve endogenous protein expression in a particular way. We chose the well-known tumor suppressors P53 and PTEN as endogenous gene targets for *in vitro* research. SgRNA spacers are complementary to sequences close to the translation start site of target mRNA (Figure 4A). The expression of each sgRNA led to a substantial activation of P53 and PTEN protein expressions two days after transient transfection in T24 and 5637 bladder cancer cells, compared to the nontarget sgRNA control protein expressions, as determined by western blot (Figure 4B and C, Supplementary Figure S4E and F). To demonstrate that dCasRx-SINEB2 functions at the post-transcriptional levels, P53 and PTEN mRNA were quantified by qRT-PCR (Figures 4D and E). Additionally, ActD was employed to inhibit transcription, and the results of the qRT-PCR experiment revealed that dCasRx-SINEB2 did not affect the rate at which P53 and PTEN mRNA degraded (Figure 4F and G), demonstrating its post-transcriptional level of action. According to these findings, the dCasRx-SINEB2 system could be a helpful tool for promoting protein expression of downstream gene.

### Targeting P53 and PTEN activation by CRISPR/dCasRx-SINEB2 regulates bladder cancer cell activities

**Figure 5.**
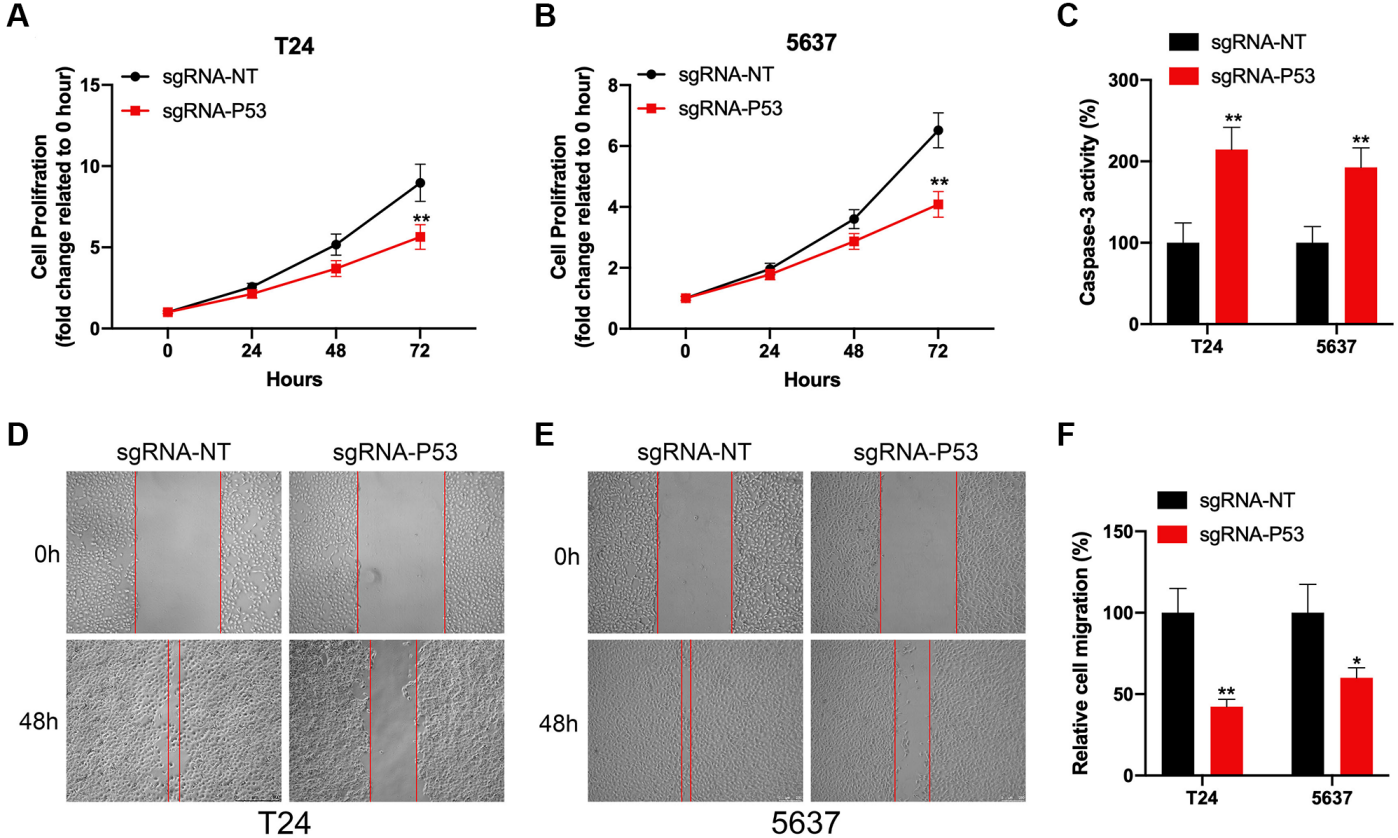
The effect of dCasRx-SINEB2-enhanced P53 expression on bladder cancer cells. (**A**, **B**) The CCK-8 experiment demonstrated the effect of dCasRx-SINEB2-activated P53 expression on the proliferation of bladder cancer cells (T24 and 5637). (**C**) Caspase-3/ELISA shows that dCasRx-SINEB2-activated P53 expression affects T24 and 5637 cell apoptosis. (**D–F**) After dCasRx-SINEB2 triggered P53, wound healing assays were utilized to assess the migratory abilities of two bladder cancer cells. (**F**) The mobility of cells was demonstrated by the difference in cell margin between 0 and 48 h; the % of the region that had healed was determined. The values are shown as means ± SD. Two-tailed *t*-test between groups: *P < 0.05; **P < 0.01.

**Figure 6.**
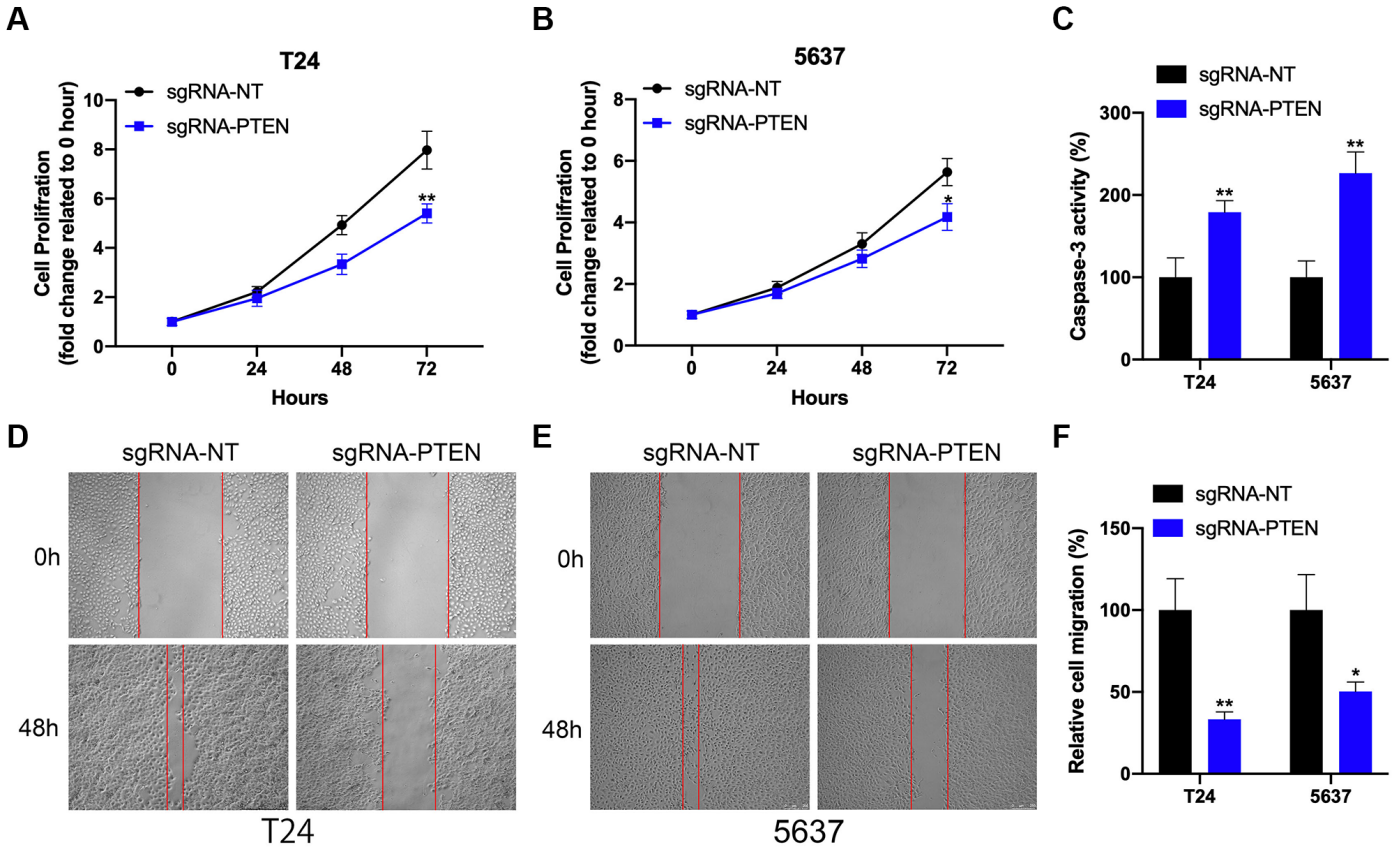
The effect of dCasRx-SINEB2-enhanced PTEN expression on bladder cancer cells. (**A, B**) The CCK-8 experiment showed the impact of dCasRx-SINEB2-activated PTEN expression on the proliferation of bladder cancer cells (T24 and 5637). (**C**) The caspase-3/ELISA assay demonstrates the impact of dCasRx-SINEB2-activated PTEN expression on cell apoptosis (T24 and 5637). (**D–F**) After dCasRx-SINEB2 activated PTEN, assays on wound healing were performed to examine the migratory capabilities of two bladder cancer cells. (F) Calculating the percentage of the healed area and observing the change in cell margin between 0–48 h revealed the cells' migration. The statistics are displayed as means ± SD. The two-tailed *t*-test between the groups was conducted. The *P < 0.05 and **P < 0.01 indicate statistical significance.

On the bladder cancer cells, the impact of P53 protein expression triggered by dCasRx-SINEB2 was evaluated. According to the CCK-8 assay results, P53 activation reduced the proliferation of bladder cancer cells (T24 and 5637) (Figures 5A and B). After that, an ELISA assay was utilized to measure the apoptosis of the bladder cancer cell. In both bladder cancer cell lines, the dCasRx-SINEB2-activated P53 expression greatly accelerated cell death (Figure 5C). Ultimately, the wound healing assay demonstrated that the P53 targeting construct dCasRx-SINEB2 decreased the migration of both bladder cancer cells (Figure 5D–F). Additionally, using a variety of functional assays, we investigated the impact of activated endogenous PTEN expression in bladder cancer cells. PTEN is a well-researched anti-oncogene that effectively regulates cellular proliferation (24). According to the cell proliferation assay results, PTEN targeting by dCasRx-SINEB2 greatly slowed the growth of both T24 and 5637 bladder cancer cells (Figures 6A and B). As a result of the dCasRx-SINEB2 targeting PTEN, it was found that both bladder cancer cells significantly more frequently underwent cell death when the cell apoptosis was examined using caspase-3 and ELISA (Figure 6C). The wound healing assay also showed that both bladder cancer cells’ migration tended to be reduced when PTEN was targeted by dCasRx-SINEB2 (Figure 6D–F). Additionlly, we also chose the well-known oncogene MYC and negative control EGFP for in vitro study. The results showed that targeting MYC activation by CRISPR/dCasRx-SINEB2 promotes tumor development and metastasis (Supplementary Figure S11), but targeting EGFP activation has no significant impact on cell mobility (Supplementary Figure S12).

### CRISPR/dCasRx-SINEB2 inhibits tumor development and metastasis *in vivo*

**Figure 7.**
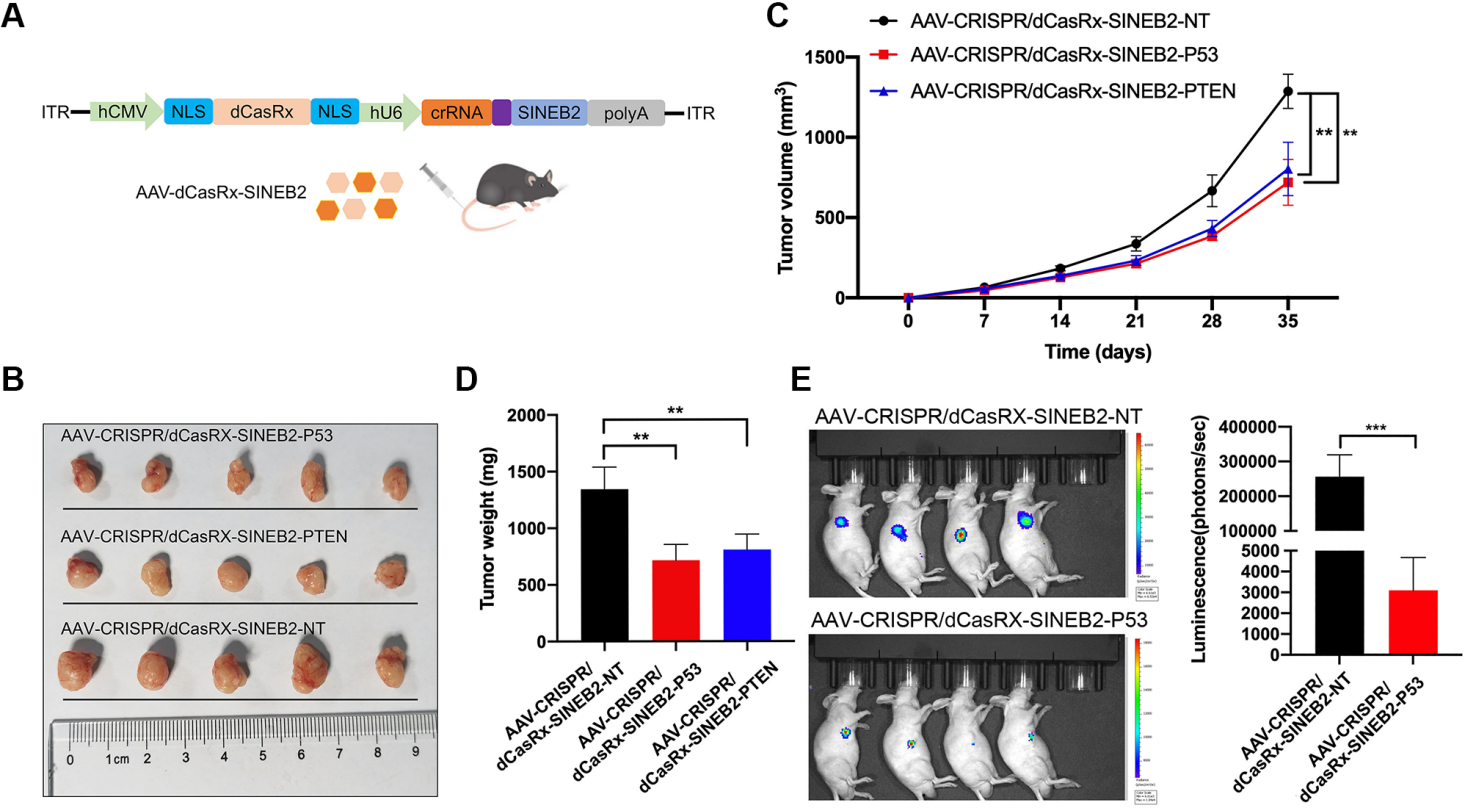
CRISPR/dCasRx-SINEB2 inhibits tumor development and metastasis *in vivo*. (**A**) Adult mice of the wild type were injected in the tail vein with dCasRx and gRNA-SINEB2 targets using an all-in-one AAV vector system. (B–D) Compared to a non-targeting control, CRISPR/dCasRx-SINEB2-P53 and CRISPR/dCasRx-SINEB2-PTEN effectively suppressed *in vivo* tumor development. (**B**) Following cell transplantation into mice, the tumor volume (**C**) and weight (**D**) were determined at the specified time points. (**E**) Measurement of a metastatic model's bioluminescence imaging. The signal intensities for luminescence are displayed. The statistics are expressed as means ± SD. Two-tailed *t*-tests across the groups: **P < 0.01; ***P < 0.001.

To provide an approach towards clinical usage, we then packed dCasRx-SINEB2 into all-in-one AAVs and performed *in vivo* tests using subcutaneous tumor models to further investigate possible uses (Figure 7A). Volumes and weights of xenografted tumors decreased compared to those in the non-targeting control group after dCasRx-SINEB2 activated PTEN and P53 proteins (Figure 7B–D). An *in vivo* bladder cancer lung metastasis model was developed utilizing the T24 cell lines that could consistently express luciferase. The dCasRx-SINEB2-P53 device greatly reduced lung metastases following tail vein injection of AAVs (Figure 7E). According to these findings, protein activation can be carefully targeted with CRISPR/dCasRx-SINEB2 to effectively treat human cancer cells.

## DISCUSSION

CRISPR-based gene regulation strategy often fuses a regulatory factor to the Cas protein, which can be used to regulate transcription or make epigenetic modifications to DNA/RNA (16,20,26,27). Nevertheless, fusion genes are often too large to be delivered *in vivo*, and sometimes overexpression of a regulatory factor can adversely affect the normal growth of host cells. Another strategy that may be adopted is to fuse a hairpin structure from noncoding RNAs to the guide RNA of the CRISPR system to facilitate the recruitment of RNA-binding proteins to perform corresponding biological functions (28–30). One of our recent studies demonstrated the feasibility of genetic manipulation by CRISPR using lncRNA functional elements (31).

The ability to regulate gene expression at the translational level would be of significant interest, not only as a tool for studying fundamental biological phenomena, but also as novel therapeutic strategy and a potential route towards biologic pharmaceutic production. Yet, while several powerful strategies exist for manipulating gene expression at the genomic or transcriptional level, few such methods exist for modulating translation (9–11). In this study, we combined the RNA-targeting sgRNA of CRISPR/CasRx with an inverted SINBE2 repeat motif to boost the translation of its target mRNA. The design of dCasRx-SINEB2 is inspired from the uchl1 lncRNA, which Carrieri et al. demonstrated to improve the translation of its equivalent sense mRNA through base-pairing and the recruitment of more ribosomes via the functional element SINEB2 (4–6). Under similar circumstances, our technique increases target protein expression just as effectively as, if not better than, the traditional RNAe method (Figures 1 and 2). Additionally, neither mRNA production (Figure 2B) nor stability were hampered by dCasRx-SINEB2 (Figure 2D). On the other hand, we show that dCasRx-SINEB2 can target mRNAs of interest that it affects in a translation-dependent way while having little off-targeting activity.

Two key advantages of dCasRx-SINEB2 over congeneric technologies are: (i) They do so without causing stable genetic changes in the target cells. (ii) They often induce the targeted protein at a higher level than most traditional gene expression enhancement techniques. This technology can increase the expression level of the exogenously introduced reporter protein from the background level to nearly 70%, and also has a several times activation efficiency for the cellular endogenous proteins. These characteristics make dCasRx-SINEB2 a potentially useful tool for several applications. First, dCasRx-SINEB2 can be employed as molecular biology reagents. While there are potential tools such as CRISPR-based artificial transcription factors to activate gene transcription, increasing the concentration of specific proteins is required in many cases where mRNA and protein levels are not always positively correlated (Supplementary Figure S10). To enhance translation, dCasRx-SINEB2 might be designed to target a single gene of interest or a tag shared by numerous targets, technically making it the opposite of siRNAs and CRISPRi/a. Second, given their impact on translation, dCasRx-SINEB2 might be used to produce proteins. More than 130 therapeutic proteins are already being used, and many more, such as antibodies, are being developed (32). Most production strategies have focused on improving culture conditions and recombinant gene transcription, leaving the potential for post-transcriptional strategies. Transiently transfected cells, notably CHO and HEK293T, have recently been used to create large-scale production platforms (33). The information provided here and elsewhere supports the viability of dCasRx-SINEB2 and its possible integration with current platforms (34).

In this study, plasmid DNA was used to transport the dCasRx-SINEB2 expression cassette into cells, where it was then generated. Such plasmid transfection could be widely used for *in vitro* applications. More importantly, another core advantage is the small size of dCasRx-SINEB2, which should permit more efficient viral delivery and gene therapy. The dCasRx-SINEB2 sequence element could potentially be incorporated into an all-in-one AAV platform for use in appropriate cell lines *in vitro* (Figure 3) or AAV-targeted cells and tissues *in vivo* (Figure 7). The dCasRx-SINEB2 also could be introduced into cells to enhance the expression of an endogenous protein (such as P53 and PTEN in Figure 4) or used in combination with another expression plasmid carrying the target protein, such as the EGFP (Figure 2), EGFP fusion proteins, and antibody heavy and light chains.

Since it complements the target mRNA at both its 5′ untranslated and translated regions, dCasRx-SINEB2 may have a very high level of specificity. In this work, dCasRx-SINEB2-EGFP and a negative control plasmid DNA were transfected into HEK293T cells that had consistent EGFP expression, and quantitative mass spectrometry was used to analyze the proteome. There was no discernible difference between these two groups, except for the increased EGFP expression in the experimental condition (Supplementary Figure S5C), demonstrating that dCasRx-SINEB2 did not affect the expression of non-targeted cellular proteins. In addition, we confirmed that HNRNPK and PTBP1 are indispensable RNA-binding proteins. These proteins led to the construction of translational initiation complexes, thus enhancing the translation of the target mRNA.

The present post-transcriptional regulation tool, which targets endogenous mRNA translation, increased the expression of the targeted proteins (PTEN and P53) while suppressing the proliferation and migration of bladder cancer cells. This result suggests that dCasRx-SINEB2 may be a general tool for protein activation and control of cellular processes. Additionally, we employed dCasRx-SINEB2 technology to induce highly targeted anti-oncogene protein production in bladder cancer cells *in vivo*. Importantly, this method prevented bladder cancer lung metastases and the growth of subcutaneously transplanted tumors in naked mice. As a result, this method can increase protein production of anti-oncogenic genes in mouse tumors and human cell lines.

In the preparation of this manuscript, an interesting work proposed a similar strategy called ‘CRISPR-RNAa’. The system achieves a similar boost in translation efficiency by fusing dCasRx directly to proteinaceous translation initiation factors, though it focuses largely on translation activation in bacteria (35). That strategy can also be considered in the future in eukaryotic mammalian cells and compared with our tool in terms of translational activation efficiency. It is certain that our tool is more suitable for integration into capacity-restricted vectors such as AAV because it uses a shorter SINEB2 translation activation element.

## CONCLUSION

Collectively, this study showed the impact of incorporating the lncRNA functional domain (SINEB2) into a CRISPR/dCasRx-sgRNA system for the first time. The dCasRx-SINEB2 technology may be a potent new tool for increasing protein expression in biological research and the production of biopharmaceuticals.

## DATA AVAILABILITY

The data underlying this article are available in the article and in its online supplementary material or will be shared on reasonable request to the corresponding author.

## Supplementary Material

gkad010_Supplemental_File
