# Supplementary material for: Enhancement of protein translation by CRISPR/dCasRx coupled with SINEB2 repeat of noncoding RNAs
Source: Nucleic Acids Res. 2023 Jan 30;51(6):e33. doi: 10.1093/nar/gkad010 (PMC10085674; doi:10.1093/nar/gkad010)
Supplement: gkad010_Supplemental_File [file gkad010_supplemental_file.docx]

**Supplementary Table.1**

**Maps of plasmids used in this study.**

| pHS-AVC-1079 | pZDonor-PGK-EGFP |
| --- | --- |
| pHS-AVC-1080 | hU6-RfxCas13d DR30-EGFP sgRNA-SINE B2-hEF1a-dRfxCas13d-NLS |
| pHS-AVC-1081 | hU6-RfxCas13d DR30-NC sgRNA-SINE B2-hEF1a-dRfxCas13d-NLS |
| pHS-AVC-1082 | hU6-RfxCas13d DR30-P53 sgRNA-SINE B2-hEF1a-dRfxCas13d-NLS |
| pHS-AVC-1083 | hU6-RfxCas13d DR30-PTEN sgRNA-SINE B2-hEF1a-dRfxCas13d-NLS |
| pHS-B-0630 | pZDonor-hU6-RfxCas13d DR30-BsaI(AAAC)-BsaI(CAGT)-SINE B2-hEF1a-RfxCas13d-NLS |
| pHS-B-0631 | pZDonor-hU6-RfxCas13d DR30-BsaI(AAAC)-BsaI(CAGT)-SINE B2-hEF1a-RfxCas13d |

**Supplementary Table.2**

Plasmid sequence

hU6-RfxCas13d DR30-BsaI(AAAC)-BsaI(CAGT)-SINE B2-hEF1a-RfxCas13d-NLS

GGCAGGAAGAGGGCCTATTTCCCATGATTCCTTCATATTTGCATATACGATACAAGGCTGTTAGAGAGATAATTAGAATTAATTTGACTGTAAACACAAAGATATTAGTACAAAATACGTGACGTAGAAAGTAATAATTTCTTGGGTAGTTTGCAGTTTTAAAATTATGTTTTAAAATGGACTATCATATGCTTACCGTAACTTGAAAGTATTTCGATTTCTTGGCTTTATATATCTTGTGGAAAGGACGAAACACCGCACTAGTGCGAATTTGCACTAGTCTAAAACTGAGACCACAGGTCTCACAGTGCTAGAGGAGGTCAGAAGAGGGCATTGGATCCCCCAGAACTGGAGTTATACGGTAACCTCGTGGTGGTTGTGAACCACCATGTGGATGGATATTGAGTTCCAAACACTGGTCCTGTGCAAGAGCATCCAGTGCTCTTAAGTGCTGAGCCATCTCTTTAGCTCCTTTTTTTAAGCTTGGCTCCGGTGCCCGTCAGTGGGCAGAGCGCACATCGCCCACAGTCCCCGAGAAGTTGTGGGGAGGGGTCGGCAATTGAACCGGTGCCTAGAGAAGGTGGCGCGGGGTAAACTGGGAAAGTGATGTCGTGTACTGGCTCCGCCTTTTTCCCGAGGGTGGGGGAGAACCGTATATAAGTGCAGTAGTCGCCGTGAACGTTCTTTTTCGCAACGGGTTTGCCGCCAGAACACAGGTAAGTGCCGTGTGTGGTTCCCGCGGGCCTGGCCTCTTTACGGGTTATGGCCCTTGCGTGCCTTGAATTACTTCCACCTGGCTGCAGTACGTGATTCTTGATCCCGAGCTTCGGGTTGGAAGTGGGTGGGAGAGTTCGAGGCCTTGCGCTTAAGGAGCCCCTTCGCCTCGTGCTTGAGTTGAGGCCTGGCCTGGGCGCTGGGGCCGCCGCGTGCGAATCTGGTGGCACCTTCGCGCCTGTCTCGCTGCTTTCGATAAGTCTCTAGCCATTTAAAATTTTTGATGACCTGCTGCGACGCTTTTTTTCTGGCAAGATAGTCTTGTAAATGCGGGCCAAGATCTGCACACTGGTATTTCGGTTTTTGGGGCCGCGGGCGGCGACGGGGCCCGTGCGTCCCAGCGCACATGTTCGGCGAGGCGGGGCCTGCGAGCGCGGCCACCGAGAATCGGACGGGGGTAGTCTCAAGCTGGCCGGCCTGCTCTGGTGCCTGGCCTCGCGCCGCCGTGTATCGCCCCGCCCTGGGCGGCAAGGCTGGCCCGGTCGGCACCAGTTGCGTGAGCGGAAAGATGGCCGCTTCCCGGCCCTGCTGCAGGGAGCTCAAAATGGAGGACGCGGCGCTCGGGAGAGCGGGCGGGTGAGTCACCCACACAAAGGAAAAGGGCCTTTCCGTCCTCAGCCGTCGCTTCATGTGACTCCACGGAGTACCGGGCGCCGTCCAGGCACCTCGATTAGTTCTCGAGCTTTTGGAGTACGTCGTCTTTAGGTTGGGGGGAGGGGTTTTATGCGATGGAGTTTCCCCACACTGAGTGGGTGGAGACTGAAGTTAGGCCAGCTTGGCACTTGATGTAATTCTCCTTGGAATTTGCCCTTTTTGAGTTTGGATCTTGGTTCATTCTCAAGCCTCAGACAGTGGTTCAAAGTTTTTTTCTTCCATTTCAGGTCCCGGGTAACTGATCATAATTCGACCCAAGTTTGTACAAAAAAGCAGGCTGATTACCGGAGAATTCCAATTGGCGGCCGCTAATACGACTCACTATAGGGAGAACCGGTGCCACCATGATCGAAAAAAAAAAGTCCTTCGCCAAGGGCATGGGCGTGAAGTCCACACTCGTGTCCGGCTCCAAAGTGTACATGACAACCTTCGCCGAAGGCAGCGACGCCAGGCTGGAAAAGATCGTGGAGGGCGACAGCATCAGGAGCGTGAATGAGGGCGAGGCCTTCAGCGCTGAAATGGCCGATAAAAACGCCGGCTATAAGATCGGCAACGCCAAATTCAGCCATCCTAAGGGCTACGCCGTGGTGGCTAACAACCCTCTGTATACAGGACCCGTCCAGCAGGATATGCTCGGCCTGAAGGAAACTCTGGAAAAGAGGTACTTCGGCGAGAGCGCTGATGGCAATGACAATATTTGTATCCAGGTGATCCATAACATCCTGGACATTGAAAAAATCCTCGCCGAATACATTACCAACGCCGCCTACGCCGTCAACAATATCTCCGGCCTGGATAAGGACATTATTGGATTCGGCAAGTTCTCCACAGTGTATACCTACGACGAATTCAAAGACCCCGAGCACCATAGGGCCGCTTTCAACAATAACGATAAGCTCATCAACGCCATCAAGGCCCAGTATGACGAGTTCGACAACTTCCTCGATAACCCCAGACTCGGCTATTTCGGCCAGGCCTTTTTCAGCAAGGAGGGCAGAAATTACATCATCAATTACGGCAACGAATGCTATGACATTCTGGCCCTCCTGAGCGGACTGGCGCACTGGGTGGTCGCTAACAACGAAGAAGAGTCCAGGATCTCCAGGACCTGGCTCTACAACCTCGATAAGAACCTCGACAACGAATACATCTCCACCCTCAACTACCTCTACGACAGGATCACCAATGAGCTGACCAACTCCTTCTCCAAGAACTCCGCCGCCAACGTGAACTATATTGCCGAAACTCTGGGAATCAACCCTGCCGAATTCGCCGAACAATATTTCAGATTCAGCATTATGAAAGAGCAGAAAAACCTCGGATTCAATATCACCAAGCTCAGGGAAGTGATGCTGGACAGGAAGGATATGTCCGAGATCAGGAAAAATCATAAGGTGTTCGACTCCATCAGGACCAAGGTCTACACCATGATGGACTTTGTGATTTATAGGTATTACATCGAAGAGGATGCCAAGGTGGCTGCCGCCAATAAGTCCCTCCCCGATAATGAGAAGTCCCTGAGCGAGAAGGATATCTTTGTGATTAACCTGAGGGGCTCCTTCAACGACGACCAGAAGGATGCCCTCTACTACGATGAAGCTAATAGAATTTGGAGAAAGCTCGAAAATATCATGCACAACATCAAGGAATTTAGGGGAAACAAGACAAGAGAGTATAAGAAGAAGGACGCCCCTAGACTGCCCAGAATCCTGCCCGCTGGCCGTGATGTTTCCGCCTTCAGCAAACTCATGTATGCCCTGACCATGTTCCTGGATGGCAAGGAGATCAACGACCTCCTGACCACCCTGATTAATAAATTCGATAACATCCAGAGCTTCCTGAAGGTGATGCCTCTCATCGGAGTCAACGCTAAGTTCGTGGAGGAATACGCCTTTTTCAAAGACTCCGCCAAGATCGCCGATGAGCTGAGGCTGATCAAGTCCTTCGCTAGAATGGGAGAACCTATTGCCGATGCCAGGAGGGCCATGTATATCGACGCCATCCGTATTTTAGGAACCAACCTGTCCTATGATGAGCTCAAGGCCCTCGCCGACACCTTTTCCCTGGACGAGAACGGAAACAAGCTCAAGAAAGGCAAGCACGGCATGAGAAATTTCATTATTAATAACGTGATCAGCAATAAAAGGTTCCACTACCTGATCAGATACGGTGATCCTGCCCACCTCCATGAGATCGCCAAAAACGAGGCCGTGGTGAAGTTCGTGCTCGGCAGGATCGCTGACATCCAGAAAAAACAGGGCCAGAACGGCAAGAACCAGATCGACAGGTACTACGAAACTTGTATCGGAAAGGATAAGGGCAAGAGCGTGAGCGAAAAGGTGGACGCTCTCACAAAGATCATCACCGGAATGAACTACGACCAATTCGACAAGAAAAGGAGCGTCATTGAGGACACCGGCAGGGAAAACGCCGAGAGGGAGAAGTTTAAAAAGATCATCAGCCTGTACCTCACCGTGATCTACCACATCCTCAAGAATATTGTCAATATCAACGCCAGGTACGTCATCGGATTCCATTGCGTCGAGCGTGATGCTCAACTGTACAAGGAGAAAGGCTACGACATCAATCTCAAGAAACTGGAAGAGAAGGGATTCAGCTCCGTCACCAAGCTCTGCGCTGGCATTGATGAAACTGCCCCCGATAAGAGAAAGGACGTGGAAAAGGAGATGGCTGAAAGAGCCAAGGAGAGCATTGACAGCCTCGAGAGCGCCAACCCCAAGCTGTATGCCAATTACATCAAATACAGCGACGAGAAGAAAGCCGAGGAGTTCACCAGGCAGATTAACAGGGAGAAGGCCAAAACCGCCCTGAACGCCTACCTGAGGAACACCAAGTGGAATGTGATCATCAGGGAGGACCTCCTGAGAATTGACAACAAGACATGTACCCTGTTCGCAAACAAGGCCGTCGCCCTGGAAGTGGCCAGGTATGTCCACGCCTATATCAACGACATTGCCGAGGTCAATTCCTACTTCCAACTGTACCATTACATCATGCAGAGAATTATCATGAATGAGAGGTACGAGAAAAGCAGCGGAAAGGTGTCCGAGTACTTCGACGCTGTGAATGACGAGAAGAAGTACAACGATAGGCTCCTGAAACTGCTGTGTGTGCCTTTCGGCTACTGTATCCCCAGGTTTAAGAACCTGAGCATCGAGGCCCTGTTCGATAGGAACGAGGCCGCCAAGTTCGACAAGGAGAAAAAGAAGGTGTCCGGCAATTCCACTAGTGGATCCGGACCTAAGAAAAAGAGGAAGGTGTAA

hU6-RfxCas13d DR30-BsaI(AAAC)-BsaI(CAGT)-SINE B2-hEF1a-RfxCas13d

GGCAGGAAGAGGGCCTATTTCCCATGATTCCTTCATATTTGCATATACGATACAAGGCTGTTAGAGAGATAATTAGAATTAATTTGACTGTAAACACAAAGATATTAGTACAAAATACGTGACGTAGAAAGTAATAATTTCTTGGGTAGTTTGCAGTTTTAAAATTATGTTTTAAAATGGACTATCATATGCTTACCGTAACTTGAAAGTATTTCGATTTCTTGGCTTTATATATCTTGTGGAAAGGACGAAACACCGCACTAGTGCGAATTTGCACTAGTCTAAAACTGAGACCACAGGTCTCACAGTGCTAGAGGAGGTCAGAAGAGGGCATTGGATCCCCCAGAACTGGAGTTATACGGTAACCTCGTGGTGGTTGTGAACCACCATGTGGATGGATATTGAGTTCCAAACACTGGTCCTGTGCAAGAGCATCCAGTGCTCTTAAGTGCTGAGCCATCTCTTTAGCTCCTTTTTTTAAGCTTGGCTCCGGTGCCCGTCAGTGGGCAGAGCGCACATCGCCCACAGTCCCCGAGAAGTTGTGGGGAGGGGTCGGCAATTGAACCGGTGCCTAGAGAAGGTGGCGCGGGGTAAACTGGGAAAGTGATGTCGTGTACTGGCTCCGCCTTTTTCCCGAGGGTGGGGGAGAACCGTATATAAGTGCAGTAGTCGCCGTGAACGTTCTTTTTCGCAACGGGTTTGCCGCCAGAACACAGGTAAGTGCCGTGTGTGGTTCCCGCGGGCCTGGCCTCTTTACGGGTTATGGCCCTTGCGTGCCTTGAATTACTTCCACCTGGCTGCAGTACGTGATTCTTGATCCCGAGCTTCGGGTTGGAAGTGGGTGGGAGAGTTCGAGGCCTTGCGCTTAAGGAGCCCCTTCGCCTCGTGCTTGAGTTGAGGCCTGGCCTGGGCGCTGGGGCCGCCGCGTGCGAATCTGGTGGCACCTTCGCGCCTGTCTCGCTGCTTTCGATAAGTCTCTAGCCATTTAAAATTTTTGATGACCTGCTGCGACGCTTTTTTTCTGGCAAGATAGTCTTGTAAATGCGGGCCAAGATCTGCACACTGGTATTTCGGTTTTTGGGGCCGCGGGCGGCGACGGGGCCCGTGCGTCCCAGCGCACATGTTCGGCGAGGCGGGGCCTGCGAGCGCGGCCACCGAGAATCGGACGGGGGTAGTCTCAAGCTGGCCGGCCTGCTCTGGTGCCTGGCCTCGCGCCGCCGTGTATCGCCCCGCCCTGGGCGGCAAGGCTGGCCCGGTCGGCACCAGTTGCGTGAGCGGAAAGATGGCCGCTTCCCGGCCCTGCTGCAGGGAGCTCAAAATGGAGGACGCGGCGCTCGGGAGAGCGGGCGGGTGAGTCACCCACACAAAGGAAAAGGGCCTTTCCGTCCTCAGCCGTCGCTTCATGTGACTCCACGGAGTACCGGGCGCCGTCCAGGCACCTCGATTAGTTCTCGAGCTTTTGGAGTACGTCGTCTTTAGGTTGGGGGGAGGGGTTTTATGCGATGGAGTTTCCCCACACTGAGTGGGTGGAGACTGAAGTTAGGCCAGCTTGGCACTTGATGTAATTCTCCTTGGAATTTGCCCTTTTTGAGTTTGGATCTTGGTTCATTCTCAAGCCTCAGACAGTGGTTCAAAGTTTTTTTCTTCCATTTCAGGTCCCGGGTAACTGATCATAATTCGACCCAAGTTTGTACAAAAAAGCAGGCTGATTACCGGAGAATTCCAATTGGCGGCCGCTAATACGACTCACTATAGGGAGAACCGGTGCCACCATGATCGAAAAAAAAAAGTCCTTCGCCAAGGGCATGGGCGTGAAGTCCACACTCGTGTCCGGCTCCAAAGTGTACATGACAACCTTCGCCGAAGGCAGCGACGCCAGGCTGGAAAAGATCGTGGAGGGCGACAGCATCAGGAGCGTGAATGAGGGCGAGGCCTTCAGCGCTGAAATGGCCGATAAAAACGCCGGCTATAAGATCGGCAACGCCAAATTCAGCCATCCTAAGGGCTACGCCGTGGTGGCTAACAACCCTCTGTATACAGGACCCGTCCAGCAGGATATGCTCGGCCTGAAGGAAACTCTGGAAAAGAGGTACTTCGGCGAGAGCGCTGATGGCAATGACAATATTTGTATCCAGGTGATCCATAACATCCTGGACATTGAAAAAATCCTCGCCGAATACATTACCAACGCCGCCTACGCCGTCAACAATATCTCCGGCCTGGATAAGGACATTATTGGATTCGGCAAGTTCTCCACAGTGTATACCTACGACGAATTCAAAGACCCCGAGCACCATAGGGCCGCTTTCAACAATAACGATAAGCTCATCAACGCCATCAAGGCCCAGTATGACGAGTTCGACAACTTCCTCGATAACCCCAGACTCGGCTATTTCGGCCAGGCCTTTTTCAGCAAGGAGGGCAGAAATTACATCATCAATTACGGCAACGAATGCTATGACATTCTGGCCCTCCTGAGCGGACTGGCGCACTGGGTGGTCGCTAACAACGAAGAAGAGTCCAGGATCTCCAGGACCTGGCTCTACAACCTCGATAAGAACCTCGACAACGAATACATCTCCACCCTCAACTACCTCTACGACAGGATCACCAATGAGCTGACCAACTCCTTCTCCAAGAACTCCGCCGCCAACGTGAACTATATTGCCGAAACTCTGGGAATCAACCCTGCCGAATTCGCCGAACAATATTTCAGATTCAGCATTATGAAAGAGCAGAAAAACCTCGGATTCAATATCACCAAGCTCAGGGAAGTGATGCTGGACAGGAAGGATATGTCCGAGATCAGGAAAAATCATAAGGTGTTCGACTCCATCAGGACCAAGGTCTACACCATGATGGACTTTGTGATTTATAGGTATTACATCGAAGAGGATGCCAAGGTGGCTGCCGCCAATAAGTCCCTCCCCGATAATGAGAAGTCCCTGAGCGAGAAGGATATCTTTGTGATTAACCTGAGGGGCTCCTTCAACGACGACCAGAAGGATGCCCTCTACTACGATGAAGCTAATAGAATTTGGAGAAAGCTCGAAAATATCATGCACAACATCAAGGAATTTAGGGGAAACAAGACAAGAGAGTATAAGAAGAAGGACGCCCCTAGACTGCCCAGAATCCTGCCCGCTGGCCGTGATGTTTCCGCCTTCAGCAAACTCATGTATGCCCTGACCATGTTCCTGGATGGCAAGGAGATCAACGACCTCCTGACCACCCTGATTAATAAATTCGATAACATCCAGAGCTTCCTGAAGGTGATGCCTCTCATCGGAGTCAACGCTAAGTTCGTGGAGGAATACGCCTTTTTCAAAGACTCCGCCAAGATCGCCGATGAGCTGAGGCTGATCAAGTCCTTCGCTAGAATGGGAGAACCTATTGCCGATGCCAGGAGGGCCATGTATATCGACGCCATCCGTATTTTAGGAACCAACCTGTCCTATGATGAGCTCAAGGCCCTCGCCGACACCTTTTCCCTGGACGAGAACGGAAACAAGCTCAAGAAAGGCAAGCACGGCATGAGAAATTTCATTATTAATAACGTGATCAGCAATAAAAGGTTCCACTACCTGATCAGATACGGTGATCCTGCCCACCTCCATGAGATCGCCAAAAACGAGGCCGTGGTGAAGTTCGTGCTCGGCAGGATCGCTGACATCCAGAAAAAACAGGGCCAGAACGGCAAGAACCAGATCGACAGGTACTACGAAACTTGTATCGGAAAGGATAAGGGCAAGAGCGTGAGCGAAAAGGTGGACGCTCTCACAAAGATCATCACCGGAATGAACTACGACCAATTCGACAAGAAAAGGAGCGTCATTGAGGACACCGGCAGGGAAAACGCCGAGAGGGAGAAGTTTAAAAAGATCATCAGCCTGTACCTCACCGTGATCTACCACATCCTCAAGAATATTGTCAATATCAACGCCAGGTACGTCATCGGATTCCATTGCGTCGAGCGTGATGCTCAACTGTACAAGGAGAAAGGCTACGACATCAATCTCAAGAAACTGGAAGAGAAGGGATTCAGCTCCGTCACCAAGCTCTGCGCTGGCATTGATGAAACTGCCCCCGATAAGAGAAAGGACGTGGAAAAGGAGATGGCTGAAAGAGCCAAGGAGAGCATTGACAGCCTCGAGAGCGCCAACCCCAAGCTGTATGCCAATTACATCAAATACAGCGACGAGAAGAAAGCCGAGGAGTTCACCAGGCAGATTAACAGGGAGAAGGCCAAAACCGCCCTGAACGCCTACCTGAGGAACACCAAGTGGAATGTGATCATCAGGGAGGACCTCCTGAGAATTGACAACAAGACATGTACCCTGTTCGCAAACAAGGCCGTCGCCCTGGAAGTGGCCAGGTATGTCCACGCCTATATCAACGACATTGCCGAGGTCAATTCCTACTTCCAACTGTACCATTACATCATGCAGAGAATTATCATGAATGAGAGGTACGAGAAAAGCAGCGGAAAGGTGTCCGAGTACTTCGACGCTGTGAATGACGAGAAGAAGTACAACGATAGGCTCCTGAAACTGCTGTGTGTGCCTTTCGGCTACTGTATCCCCAGGTTTAAGAACCTGAGCATCGAGGCCCTGTTCGATAGGAACGAGGCCGCCAAGTTCGACAAGGAGAAAAAGAAGGTGTCCGGCAATTCC

PGK-EGFP

AATTCCACGGGGTTGGGGTTGCGCCTTTTCCAAGGCAGCCCTGGGTTTGCGCAGGGACGCGGCTGCTCTGGGCGTGGTTCCGGGAAACGCAGCGGCGCCGACCCTGGGTCTCGCACATTCTTCACGTCCGTTCGCAGCGTCACCCGGATCTTCGCCGCTACCCTTGTGGGCCCCCCGGCGACGCTTCCTGCTCCGCCCCTAAGTCGGGAAGGTTCCTTGCGGTTCGCGGCGTGCCGGACGTGACAAACGGAAGCCGCACGACTCACTAGTACCCTCGCAGACGGACAGCGCCAGGGAGCAATGGCAGCGCGCCGACCGCGATGGGCTGTGGCCAATAGCGGCTGCTCAGCAGGGCGCGCCGAGAGCAGCGGCCGGGAAGGGACGGTGCGGGAGGCGGGGTGTGGGGCGGTAGTGTGGGCCCTGTTCCTGCCCGCGCGGTGTTCCGCATTCTGCAAGCCTCCGGAGCGCACGTCGGCAGTCGGCTCCCTCGTTGACCGAATCACCGACCTCTCTCCCCAGGGGGATCCACCGGTTTGTCGACAAGCTTGGTGGCGGCTTAACTAGTTAAGGGCCCGGCGCGCCTAAGGTACCCCCGGGTAACTGATCATAATTCGACCCAAGTTTGTACAAAAAAGCAGGCTGATTACCGGTCGCCACCATGGTGAGCAAGGGCGAGGAGCTGTTCACCGGGGTGGTGCCCATCCTGGTCGAGCTGGACGGCGACGTAAACGGCCACAAGTTCAGCGTGTCCGGCGAGGGCGAGGGCGATGCCACCTACGGCAAGCTGACCCTGAAGTTCATCTGCACCACCGGCAAGCTGCCCGTGCCCTGGCCCACCCTCGTGACCACCCTGACCTACGGCGTGCAGTGCTTCAGCCGCTACCCCGACCACATGAAGCAGCACGACTTCTTCAAGTCCGCCATGCCCGAAGGCTACGTCCAGGAGCGCACCATCTTCTTCAAGGACGACGGCAACTACAAGACCCGCGCCGAGGTGAAGTTCGAGGGCGACACCCTGGTGAACCGCATCGAGCTGAAGGGCATCGACTTCAAGGAGGACGGCAACATCCTGGGGCACAAGCTGGAGTACAACTACAACAGCCACAACGTCTATATCATGGCCGACAAGCAGAAGAACGGCATCAAGGTGAACTTCAAGATCCGCCACAACATCGAGGACGGCAGCGTGCAGCTCGCCGACCACTACCAGCAGAACACCCCCATCGGCGACGGCCCCGTGCTGCTGCCCGACAACCACTACCTGAGCACCCAGTCCGCCCTGAGCAAAGACCCCAACGAGAAGCGCGATCACATGGTCCTGCTGGAGTTCGTGACCGCCGCCGGGATCACTCTCGGCATGGACGAGCTGTACAAGTAA

**Supplementary Table.3 sgRNA sequences**

| NC sgRNA | TGACGCTTAACGACTGTTATGT |
| --- | --- |
| EGFP sgRNA | CGCCCTTGCTCACCATGGTGGC |
| P53 sgRNA | ATCATCCATTGCTTGGGACGG |
| PTEN sgRNA | ATGGCTGTCATGTCTGGGAGC |


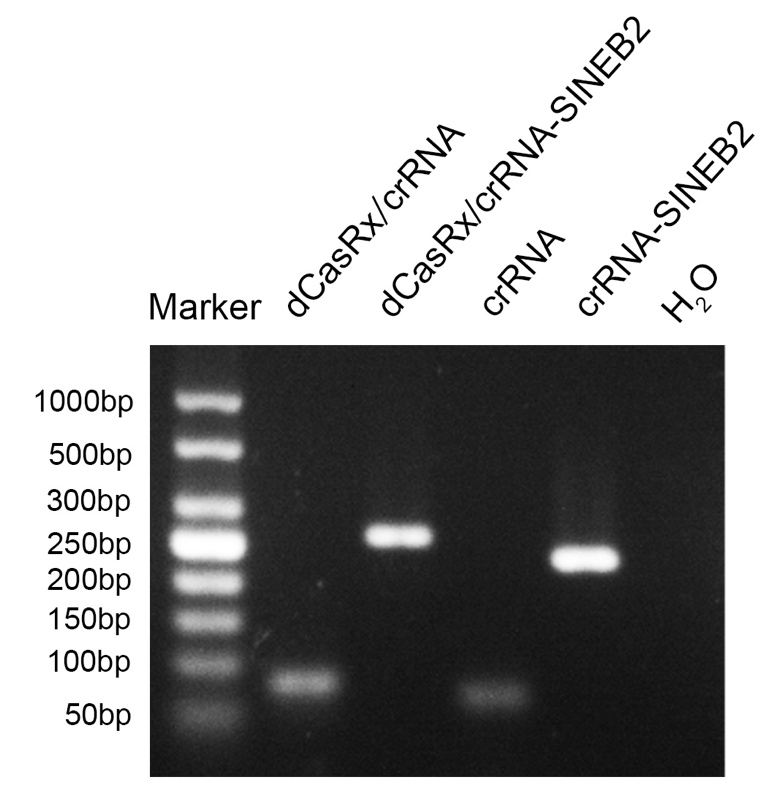


**Supplementary Figure S1. The formation of the CRISPR/dCasRx-SINEB2 system.** In this result, cellular RNAs were extracted from cells transfected with CRISPR-dCasRx/crRNA, CRISPR-dCasRx/crRNA-SINEB2, crRNA and crRNA-SINEB2 expression vectors, respectively, and were then reverse transcribed into cDNAs. Different primers were designed to amplify the corresponding cDNA regions of the guide RNAs of different constructs, including dCasRx/crRNA, dCasRx/crRNA-SINEB2, crRNA and crRNA-SINEB2. The crRNA-SINEB2 fusion element we designed could be completely expressed and existed in cells regardless of the co-expression of dCasRx protein.

**
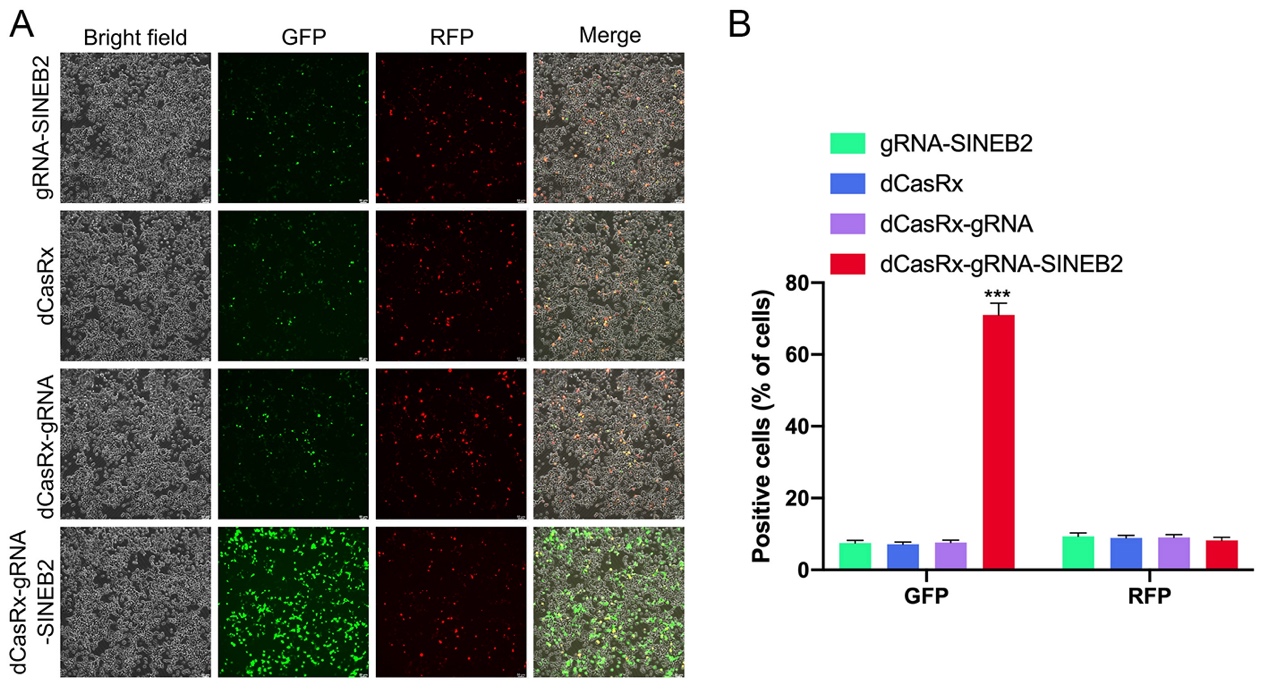
**

**Supplementary Figure S2. Enhancement of EGFP level by different constructs. (A)** The impact of dCasRx-gRNA-SINEB2 was evaluated by fluorescence spectroscopy in HEK293T cells co-transfected with pEGFP-C1, pRFP and gRNA-SINEB2 plasmid, dCasRx plasmid, dCasRx-gRNA or dCasRx-gRNA-SINEB2 plasmid. **(B)** The results depicted a flow cytometry analysis of plasmid-transfected EGFP-positive and RFP-positive cells. Statistical data from independent triplicate experiments are shown as mean±SD. The statistical significance was defined as ***P<0.001.

**
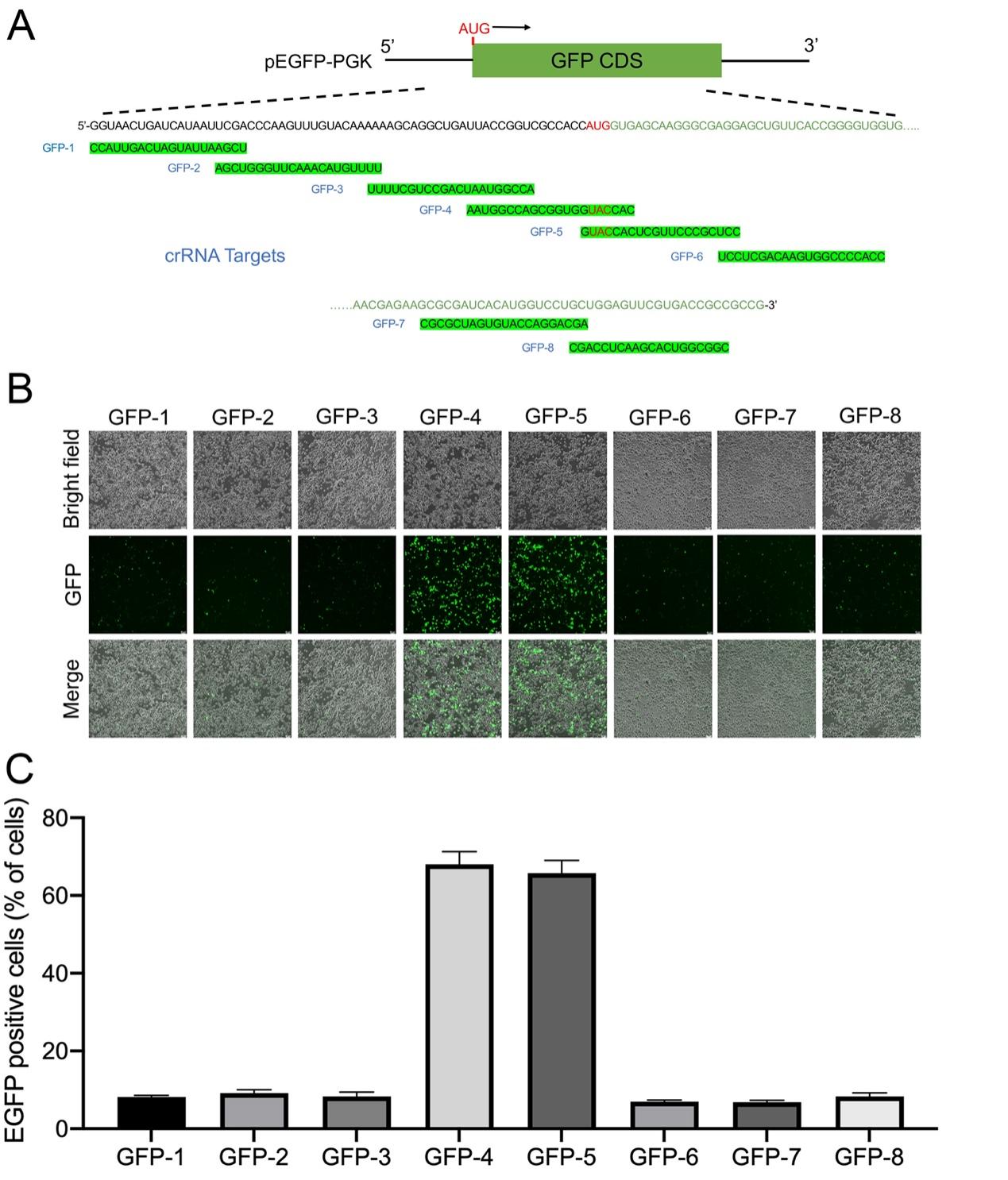
**

**Supplementary Figure S3. Enhancement of EGFP level by different gRNAs. (A)** Different target locations of the crRNAs across the GFP mRNA. **(B-C)** The effect of changing the targeting position of crRNA in GFP mRNA.

**
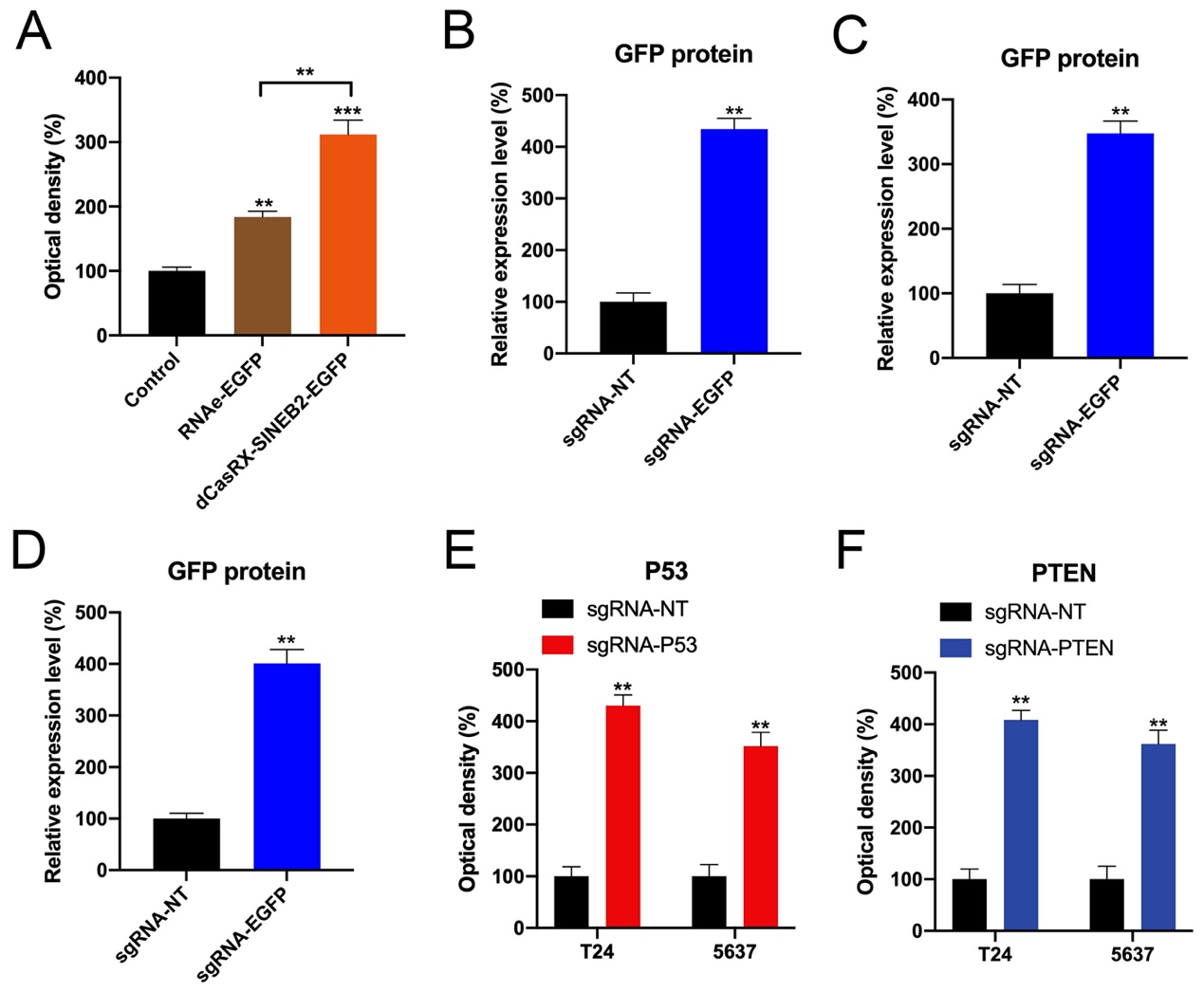
**

**Supplementary Figure S4. Protein quantification in figures. (A)** Protein quantification in Figure 2A. **(B)** Protein quantification in Figure 3A. **(C)** Protein quantification in Figure 3B. **(D)** Protein quantification in Figure 3C. **(E)** Protein quantification in Figure 4B. **(F)** Protein quantification in Figure 4C.


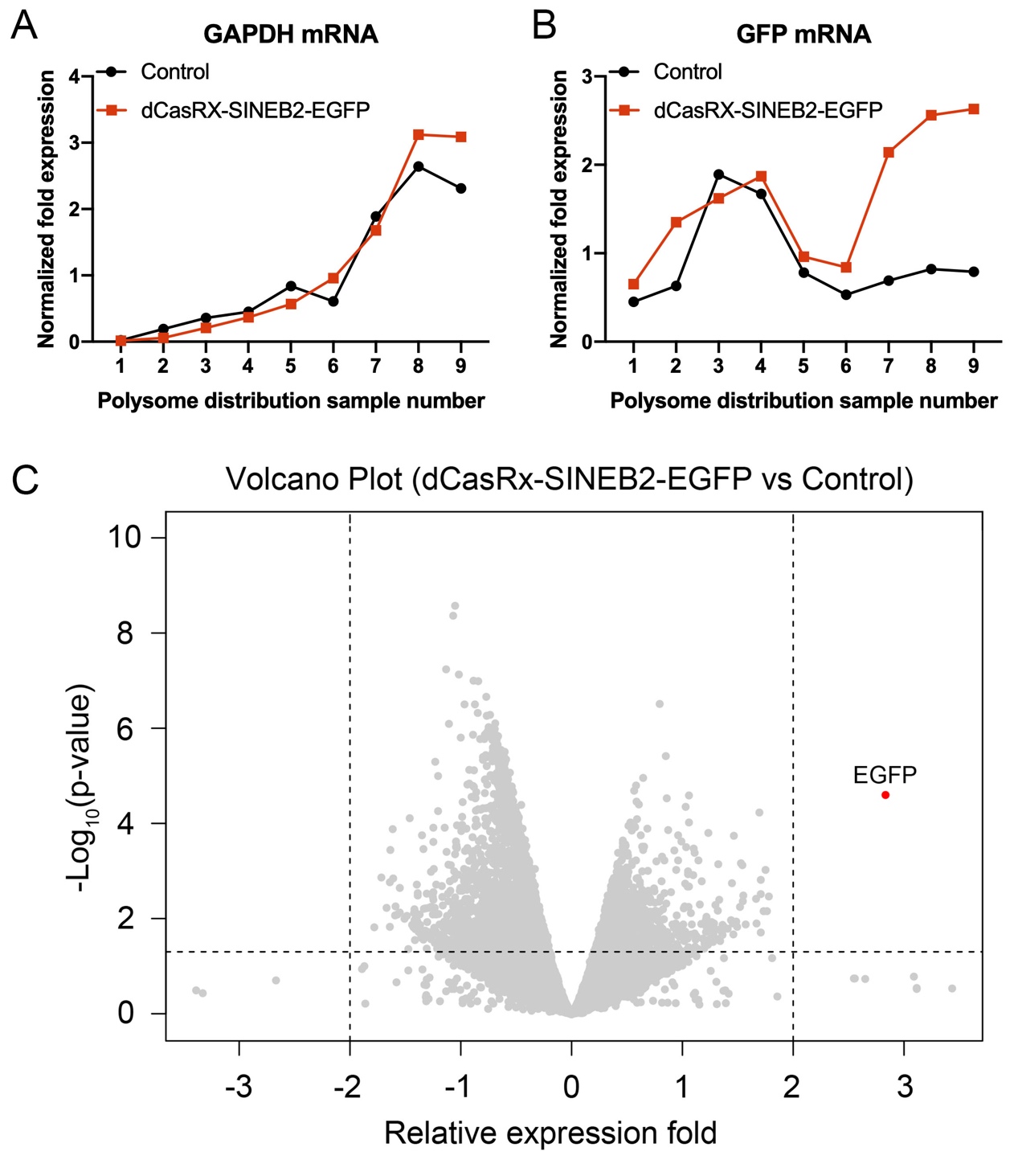


**Supplementary Figure S5. The mechanism of dCasRx-SINEB2.** (**A-B**) The distribution of the mRNA for EGFP in polysomes in HEK293T cells (**B**), with GAPDH serving as an internal control (**A**). (**C**) Proteomics analysis ensures the specificity of dCasRx-SINEB2.


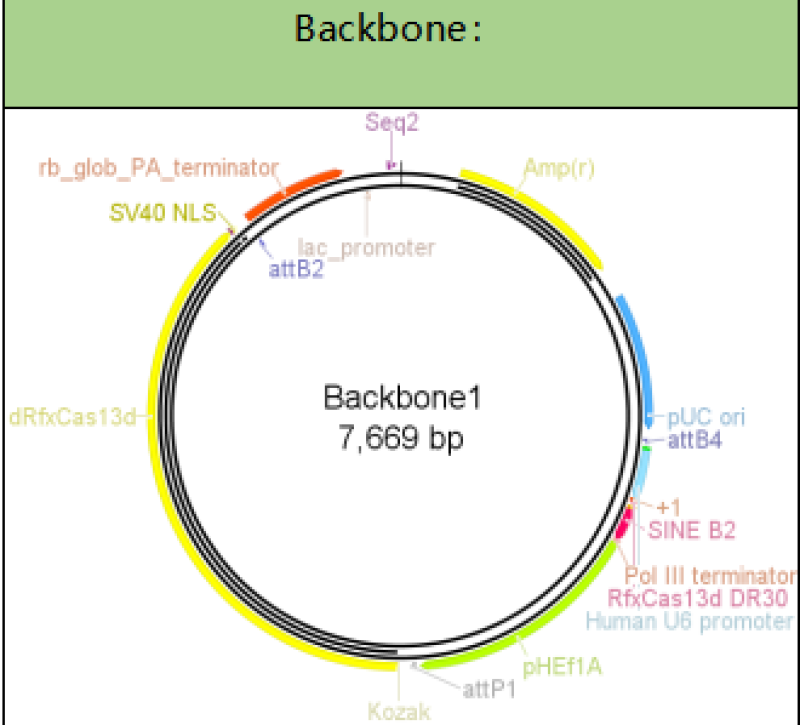


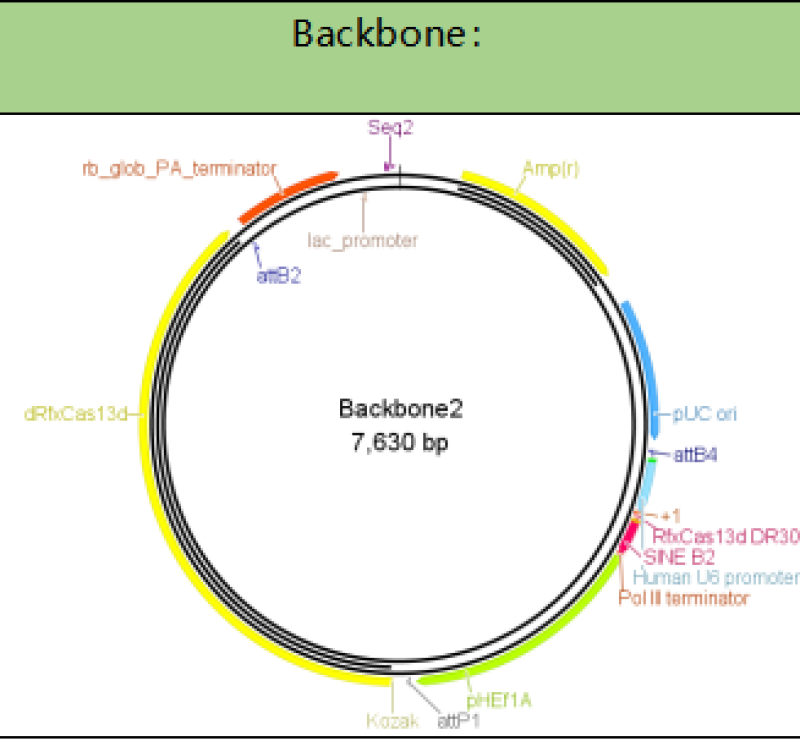


**Supplementary Figure S6. The backbone of constructs.**


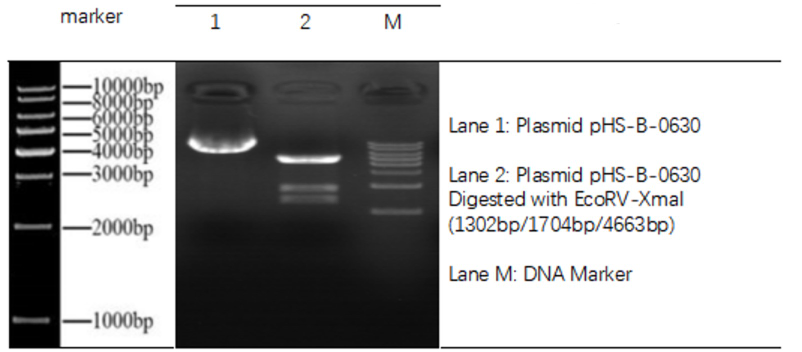


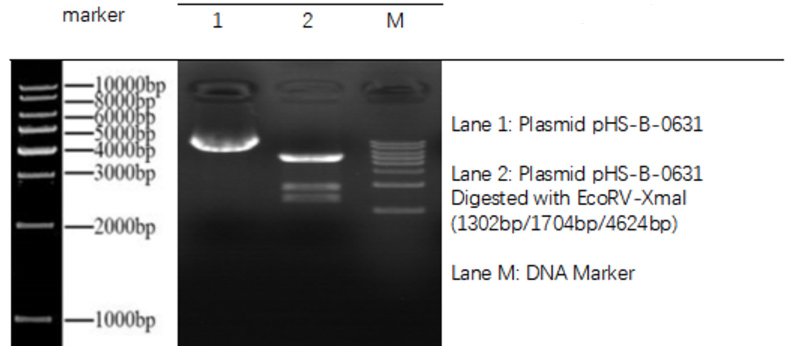


**Supplementary Figure S7. Verification of plasmid digestion.**


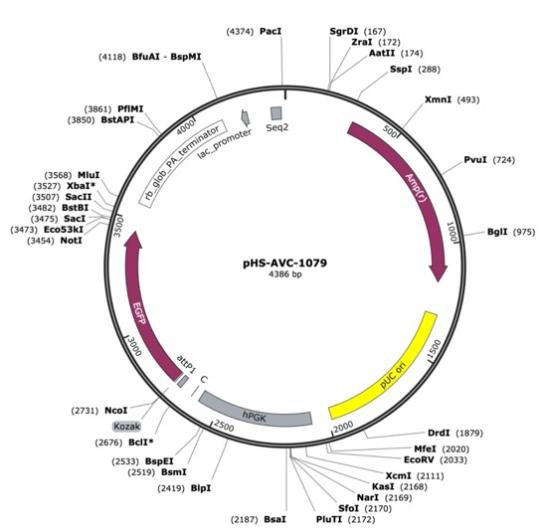

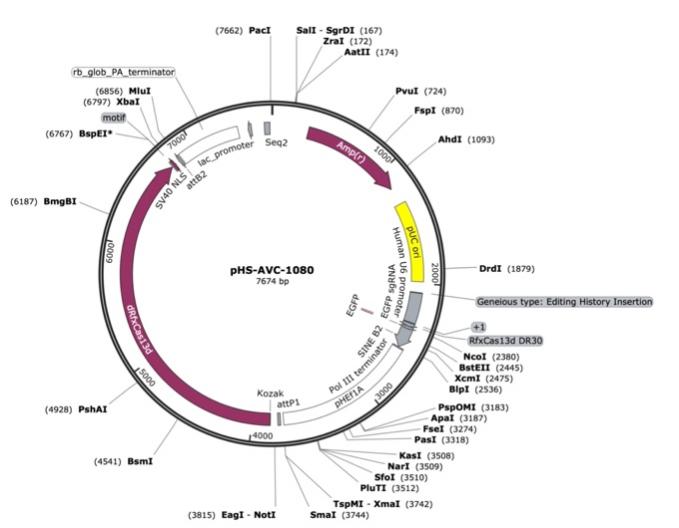


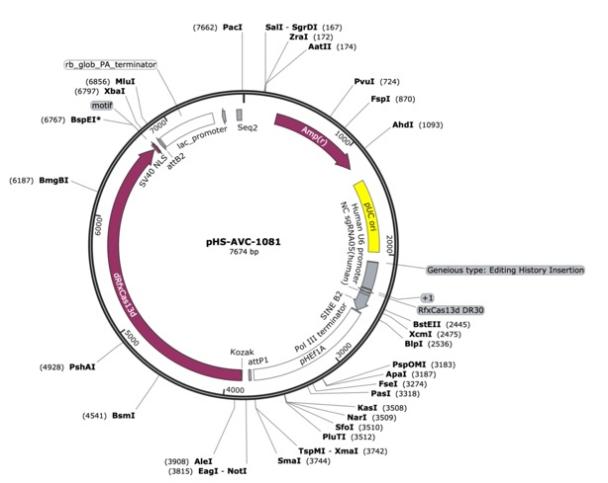

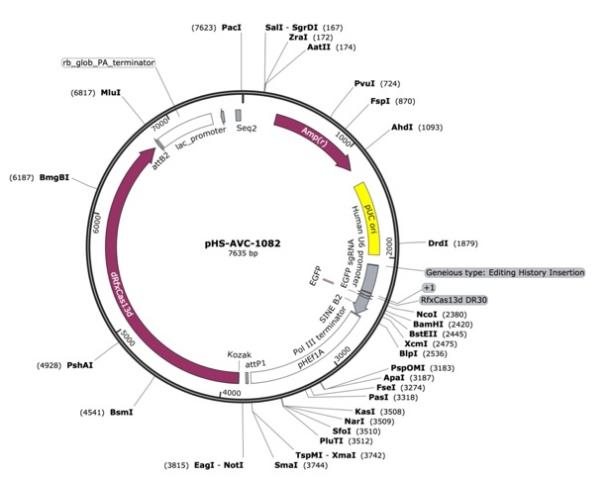


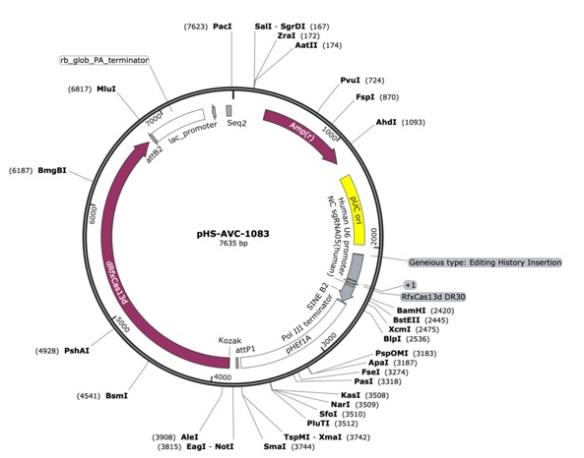

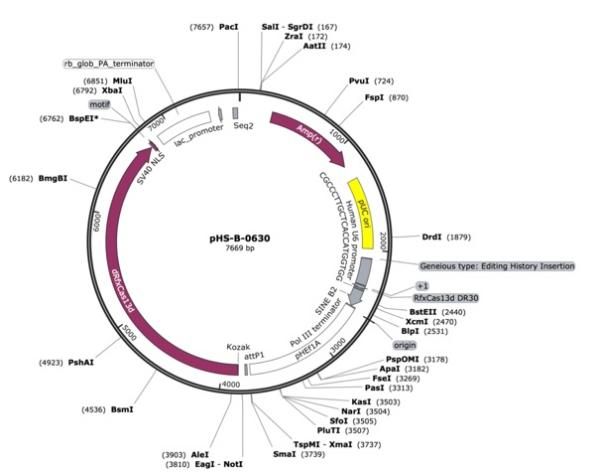


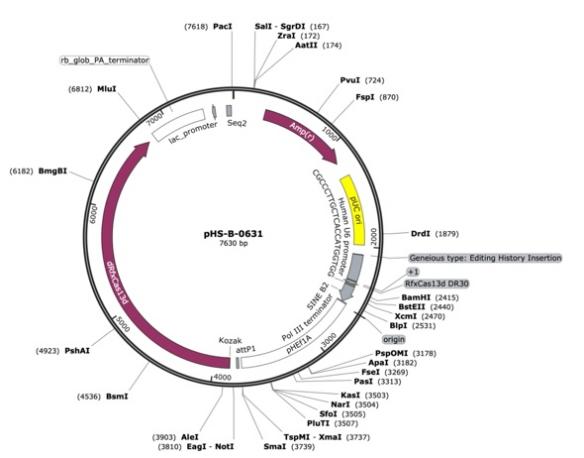


**Supplementary Figure S8. The detailed profile of vectors.**

**
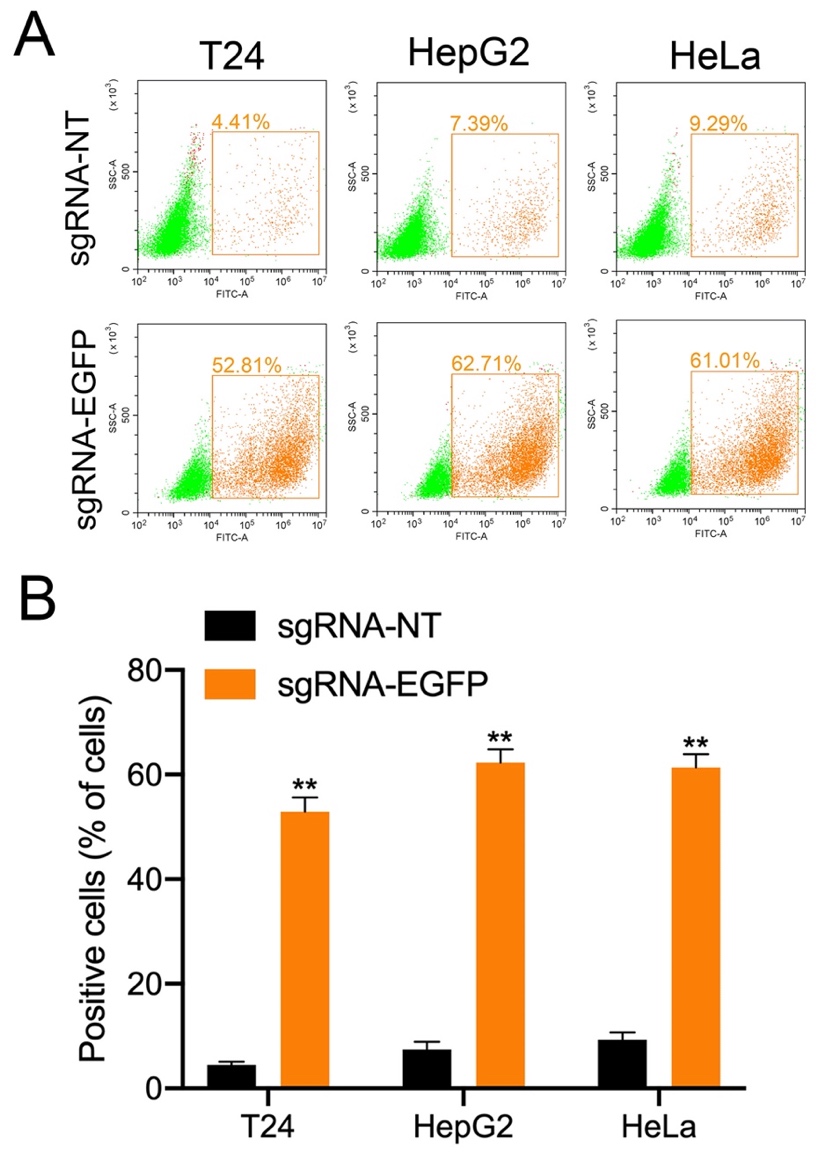
**

**Supplementary Figure S9. Enhancement of EGFP level by dCasRx-SINEB2 in different cells. (A-B)** The impact of dCasRx-SINEB2 was evaluated by flow cytometry in T24, HepG2 and HeLa cells co-transfected with pEGFP-C1 and sgRNA-NT or sgRNA-EGFP plasmid. Statistical data from independent triplicate experiments are shown as mean±SD. The statistical significance was defined as **P<0.01.


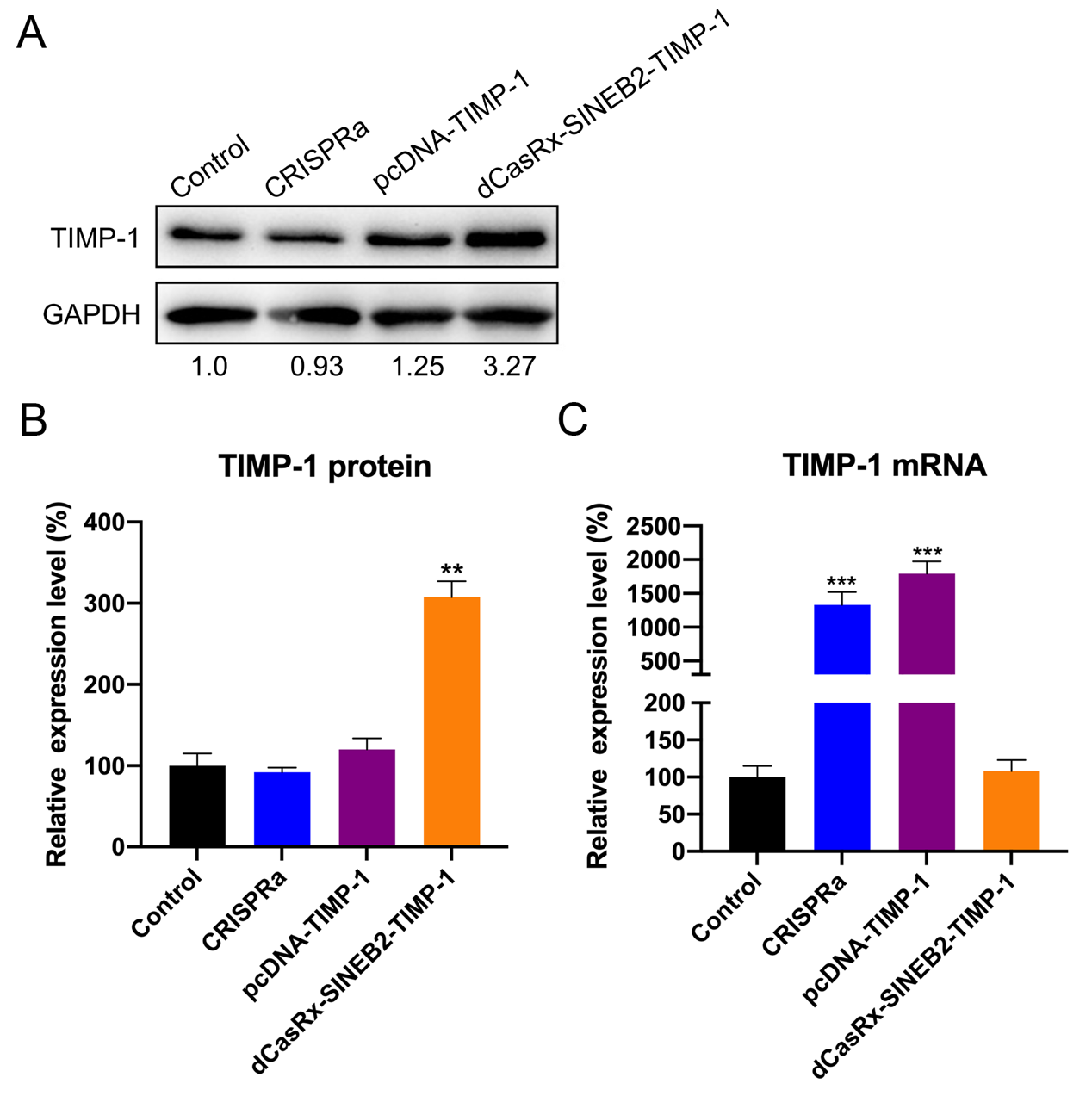


**Supplementary Figure S10. Comparison of mRNA and protein levels of TIMP-1 after treated with various tools. (A, B)** The protein expression levels of TIMP-1 in CRISPRa, pcDNA-TIMP-1 and CRISPR/dCasRx-SINEB2-TIMP-1 groups. **(C)** The mRNA expression levels of TIMP-1 in CRISPRa, pcDNA-TIMP-1 and CRISPR/dCasRx-SINEB2-TIMP-1 groups.


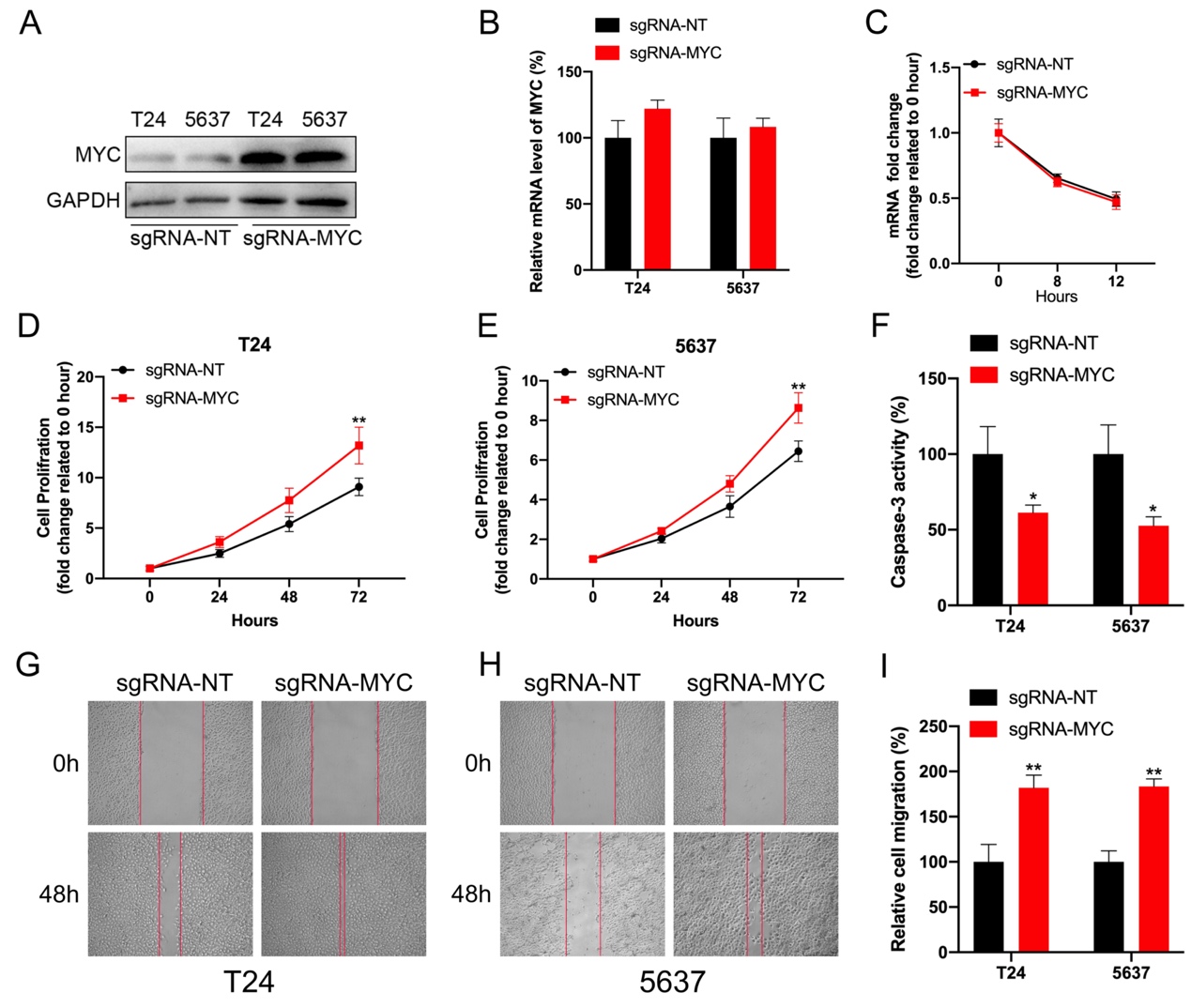


**Supplementary Figure S11. The effect of dCasRx-SINEB2-enhanced MYC expression on bladder cancer cells.** (**A, B**) MYC protein (A) and mRNA (B) expression levels were detected after being transfected with dCasRx-SINEB2-NT, dCasRx-SINEB2-P53, or dCasRx-SINEB2-PTEN in T24 and 5637 bladder cancer cells. (**C**) Actinomycin D (ActD) was applied to the bladder cell culture medium for 0, 8, or 12 hours after transfection with dCasRx-SINEB2-NT or dCasRx-SINEB2-MYC to inhibit mRNA synthesis. The qRT-PCR assay revealed that dCasRx-SINEB2 did not affect MYC mRNA stability. Data are representative of >3 independent replicates. (**D, E**) The CCK-8 experiment showed the impact of dCasRx-SINEB2-activated MYC expression on the proliferation of bladder cancer cells (T24 and 5637). (**F**) The caspase-3/ELISA assay demonstrates the impact of dCasRx-SINEB2-activated MYC expression on cell apoptosis (T24 and 5637). (**G-H**) After dCasRx-SINEB2 activated MYC, assays on wound healing were performed to examine the migratory capabilities of two bladder cancer cells. (**I**) Calculating the percentage of the healed area and observing the change in cell margin between 0-48 hours revealed the cells' migration. The statistics are displayed as means ±SD. The two-tailed *t-test* between the groups was conducted. The *P <0.05 and **P<0.01 indicate statistical significance.


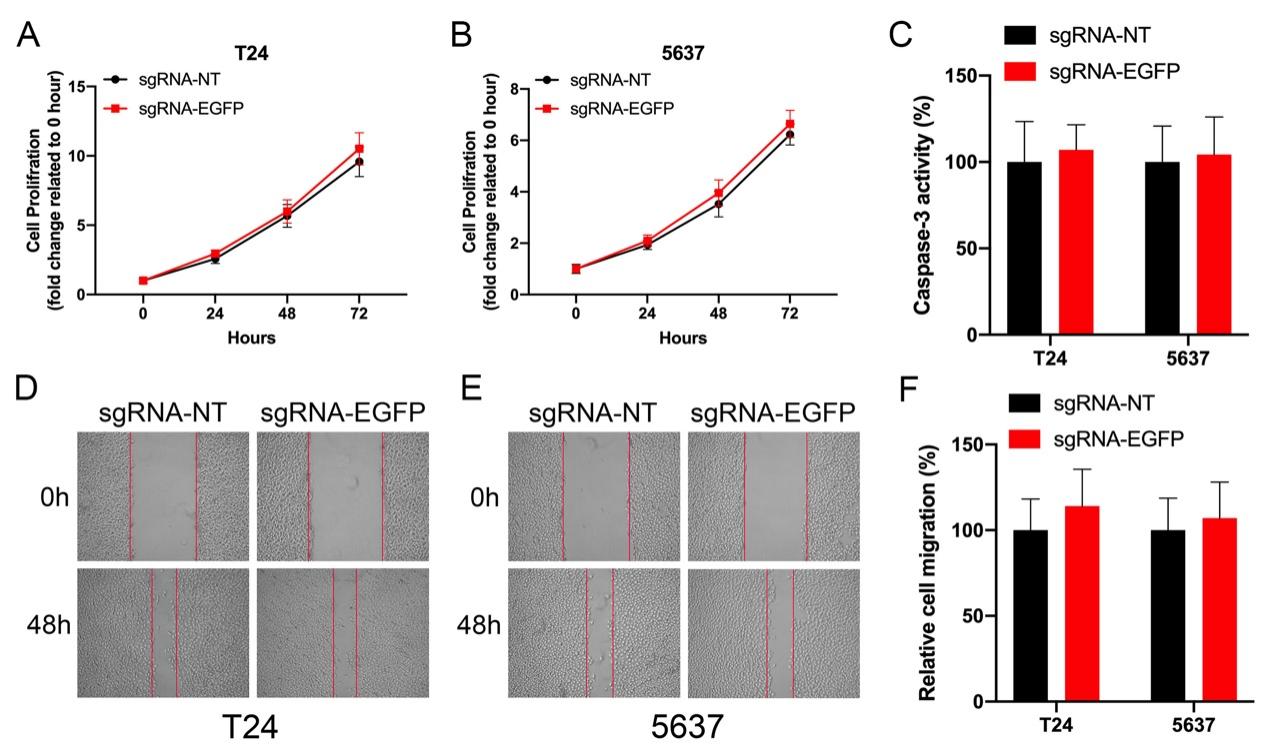


**Supplementary Figure S12. The effect of dCasRx-SINEB2-enhanced EGFP expression on bladder cancer cells.** (**A, B**) The CCK-8 experiment showed the impact of dCasRx-SINEB2-activated EGFP expression on the proliferation of bladder cancer cells (T24 and 5637). (**C**) The caspase-3/ELISA assay demonstrates the impact of dCasRx-SINEB2-activated EGFP expression on cell apoptosis (T24 and 5637). (**D-E**) After dCasRx-SINEB2 activated EGFP, assays on wound healing were performed to examine the migratory capabilities of two bladder cancer cells. (**F**) Calculating the percentage of the healed area and observing the change in cell margin between 0-48 hours revealed the cells' migration. The statistics are displayed as means ±SD. The two-tailed *t-test* between the groups was conducted. The *P <0.05 and **P<0.01 indicate statistical significance.
